# Tuning of the photophysical and electrochemical properties of symmetric and asymmetric conjugated thiophenoazomethines†

**DOI:** 10.1039/c8ra00570b

**Published:** 2018-04-03

**Authors:** Shengzhen Liu, Ti Wu, Qi Zhu, Jialing Pu, Guangxue Chen, Weimin Zhang, Zhongxiao Li

**Affiliations:** State Key Laboratory of Pulp and Paper Engineering, South China University of Technology China; Information Recording Material Lab, Lab of Printing & Packing Materials and Technology, Beijing Institute of Graphic Communication China wuti@bigc.edu.cn

## Abstract

A synthetic route towards symmetric and asymmetric thiophenoazomethines was accomplished by reaction of the readily available amine with various aldehydes. Investigation of a series of thiophenoazomethines obtained by this method indicates that the terminal groups and the degree of conjugation have a great effect on the electronic absorption and energy levels of the conjugated compounds, particularly the effect of terminal groups. The terminal withdrawing and donating groups of thiophenoazomethines led to the formation of an electronic push–pull, push–push and pull–pull system, which can perturb the electronic transitions between the ground and excited states. The flexible chain substituents on the thiophene units, which improve its solubility, also result in bathochromic absorption, but have limited effect on the energy level.

## Abstract 2

A synthetic route towards symmetric and asymmetric thiophenoazomethines was accomplished by reaction of the readily available amine with various aldehydes.

## Graphical abstract

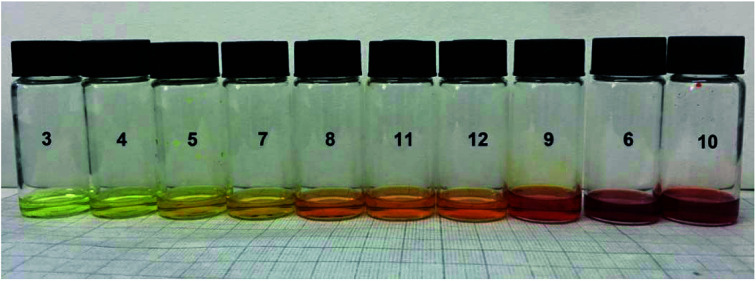

## Introduction

For more than a decade, a growing number of researchers have poured attention into conjugated materials. Because of their unique optical, electrical, and mechanical properties, these materials have gained attention for their applications in areas such as organic field-effect transistors (OFETs), organic light-emitting diodes (OLEDs) and photovoltaic cells.^1–6^ The conjugated materials are attractive candidates owing to their physical, electrical, and mechanical properties; however, their synthesis is challenging. The methods to obtain conjugated materials such as Suzuki and Wittig^7,8^ reactions require stringent reaction conditions, such as inert atmosphere, strictly anhydrous solvent and complicated post-processing.^9–11^ Hence, novel and cost-effective methods toward conjugated materials are essential and attractive.

Azomethines (–C=N–) seem to be ideal alternatives to traditional coupling methods for the synthesis of conjugated materials. First, the properties of azomethines are isoelectronic to their carbon analogues.^12,13^ Second, we should note that the synthesis of azomethines through the simple condensation reaction between an amine and an aldehyde requires less difficult purification steps and mild reaction conditions. Finally, the residual metal contamination in conventional conjugated material synthesis is nonexistent, which may lead to inconsistent physical properties in the final products.^14–16^

Despite possessing properties for device usage, azomethine conjugated materials cannot satisfy the performance demands for commercial application^17–19^. There are several problems with homoaryl azomethines such as poor solubility, irreversible radical cation formation, and undesired oxidative decomposition.^20–22^ Hence, we introduced thiophenes into the azomethine conjugated materials not only owing to their low oxidation potential, reversible oxidization, equally low band-gap properties and the advantages of easy modification,^23,24^ but also because the spectroscopic and electrochemical properties of thiophenes are compatible with functional devices.^25,26^ Considering that the tuning of the HOMO–LUMO energy gap exerts a great influence on optoelectronic properties, solid-state and charge-transport characteristics of molecular devices, we become interested in the incorporation of different groups into the thiopheneazomethines and then synthesizing the corresponding symmetric and asymmetric thiophenoazomethines and evaluating their effect on the photophysical and electrochemical properties. This research provides crucial information for the future design and synthesis of all-thiopheneazomethines with desired characteristics.

Herein, we report the design, synthesis and properties of symmetric and asymmetric conjugated thiophenoazomethines. All the compounds were synthesized by modifying the reaction conditions and the equiv. of aldehyde precursors. It is our goal to synthesize the compounds and investigate the effect of the degree of conjugation (the number of azomethine bonds) and terminal groups on the photophysical and electrochemical properties of the as-prepared conjugated compounds. Thus, the structural, photophysical, and electrochemical properties of these symmetric and asymmetric conjugated thiophenoazomethines were explored.

## Results and discussion

### Synthesis of symmetric and asymmetric conjugated thiophenoazomethines

**Scheme 1:**
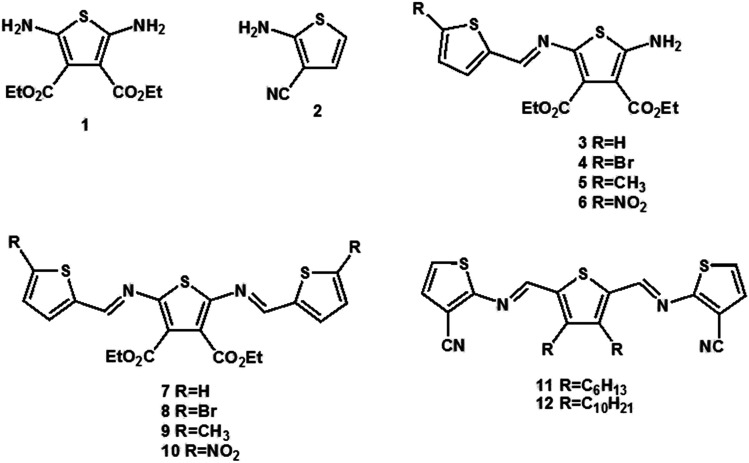
The structures of conjugated thiophenoazomethine compounds.

Symmetric and asymmetric conjugated thiophenoazomethines were obtained by simple dehydration between amine and aldehyde groups *via* hydroscopic solvents (*i.e.* anhydrous ethanol/isopropanol/butyl alcohol, anhydrous toluene and THF). We synthesized a series of conjugated thiophenoazomethines through the judicious choice of solvent, temperature and reagent stoichiometry; the structures of the compounds are as follows (Scheme 1).

**Scheme 2:**
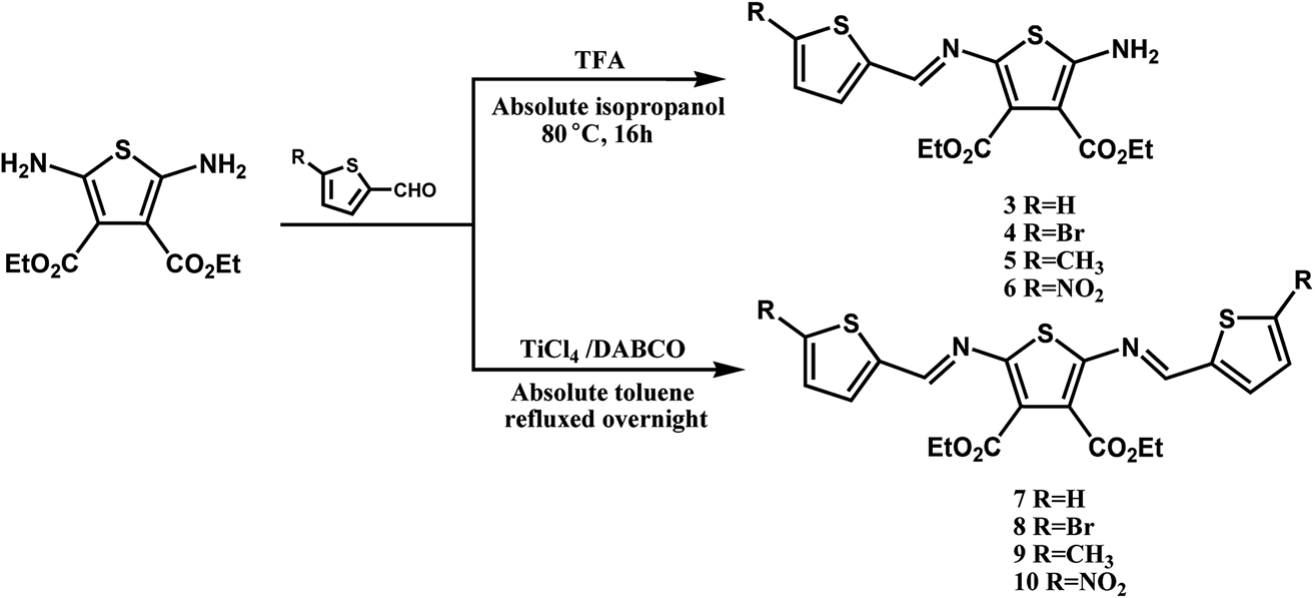
Synthesis of thiophenoazomethines compounds 3–10.

The electron withdrawing cyano group and ester group deactivate the amine and result in reduced reactivity towards the aldehyde, thus increasing the stability of 1 and 2 (ref. 27 and 28) under normal atmospheric conditions. Hence, the asymmetric conjugated thiophenoazomethines 3–6 are formed exclusively in absolute isopropanol regardless of the aldehyde used and the stoichiometry employed (Scheme 2). In the asymmetric compounds, the electron withdrawing effect of azomethine bonds further reduce the reactivity of amine. On using only higher boiling point solvents such as *n*-butanol and on prolonged heating, the reaction can be accelerated to afford the corresponding symmetric conjugated thiophenoazomethine 7. However, for the synthesis of 8–10, Lewis acids such as TiCl_4_ should be used as catalysts to decrease the reaction time while improving the yields of conjugated thiophenoazomethines. The syntheses of 3 and 7 were performed according to previously reported procedures.^27,28^

**Scheme 3:**
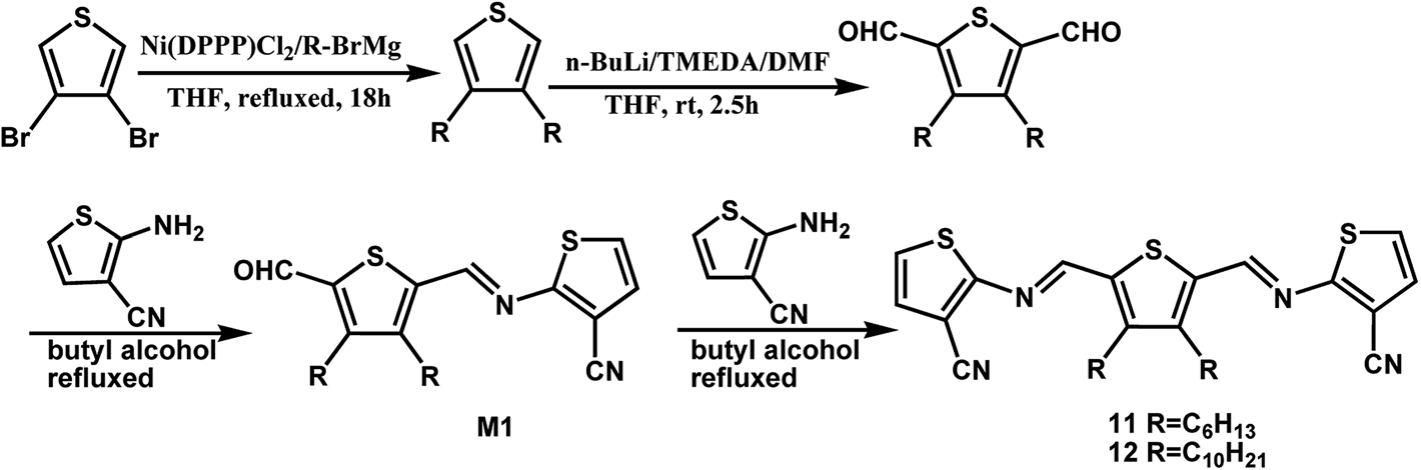
Synthesis of thiophenoazomethines compounds 11 and 12.

By changing the position of carbon and nitrogen atoms, we designed the compounds 11 and 12. In order to increase the solubility of the conjugated compounds, flexible chains were introduced on the thiophene aldehyde derivative. Condensation can be performed sequentially in a one-pot fashion directly from aldehyde derivative with two equivalents of compound 2 (Scheme 3). The thiophenealdehyde derivative in the solvent initially undergoes condensation with one equivalent of 2 to obtain intermediate M1. Moreover, the electron withdrawing effect of the cyano group to intermediate M1 increases the aldehyde reactivity towards nucleophilic addition. When an additional equivalent of 2 was added, we could easily afford the product 11 or 12. The reaction time significantly decreased from 125 hours (compound 7) to 48 hours (compounds 11 or 12) and the yield increased from 35% (compound 7) to 77% (compounds 11 or 12).

### Photophysical properties

**Fig. 1:**
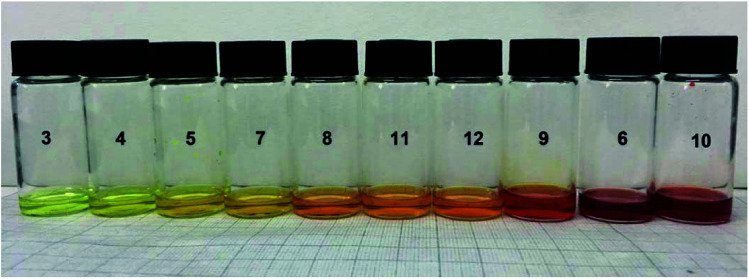
Photograph of conjugated compounds 3–12 in trichloromethane (5 × 10^−4^ M).

**Fig. 2:**
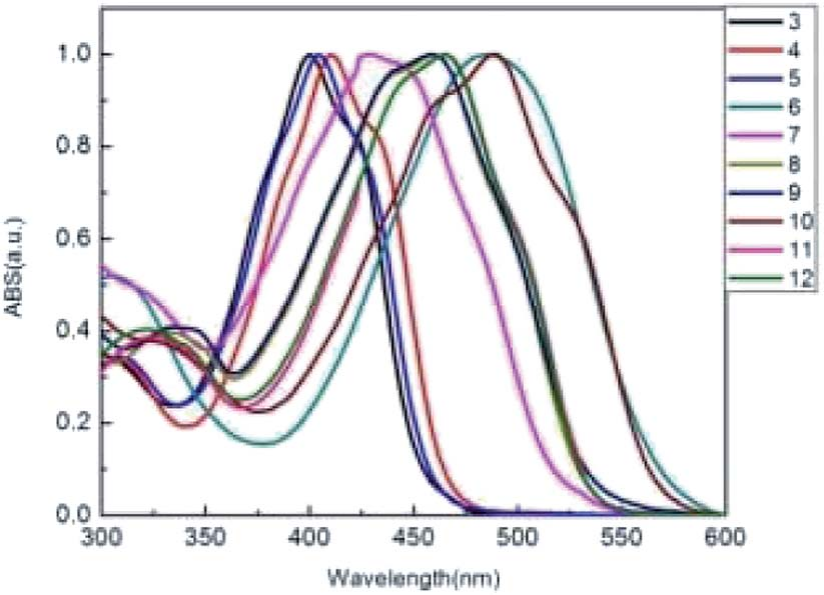
UV-visible absorption spectra of compounds 3–12 in trichloromethane.

A photograph of conjugated thiophenoazomethine compounds 3–12 in trichloromethane in daylight is shown in Fig. 1. The electronic absorption spectra of asymmetric compounds 3–6 and symmetric compounds 7–12 were recorded in trichloromethane at room temperature as shown in Fig. 2.

The extent of oligomerization and the interaction between the terminal functional groups can visually be followed by the color change as shown in Fig. 1.

The electronic absorption spectra of the conjugated thiophenoazomethines 3–12 in trichloromethane exhibit broad bands. The absorption at the shorter wavelengths is relatively weak, correspond to the π–π* transition of the conjugated thiophenes, while the strong absorption bands at the longer wavelengths correspond to an intramolecular charge transfer (ICT). As shown in Fig. 2 the absorption band of the asymmetric conjugated thiophenoazomethine 4 exhibits a slight bathochromic shift relative to the reference compound 3. The incorporation of withdrawing group –Br leads to an electronic push–pull system between the terminal withdrawing group –Br and the donating group –NH_2_ of compound 4. The push–pull action perturbs the electronic transitions and then reduces the HOMO–LUMO energy gap between the ground and excited states, which lead to the bathochromic absorption. In order to certify the above conclusion, we synthesized compound 6, which has a strong electron-deficient group –NO_2_. Not surprisingly, the strong electronic push–pull system leads to greater bathochromic absorption shifts of about 86 nm relative to compound 3. Only slight bathochromic absorption shifts are observed with 5 because of the electronic push–push system between the donating groups –CH_3_, –NH_2_ of compound 5.

Because of the existence of the conjugative effect, the same bathochromic trend with the addition of each thiophene unit was also observed in the absorption spectra. The terminal groups –Br and –NO_2_ also influence the spectroscopic properties as these electron-deficient groups of compounds 8 and 10 cause 32 and 61 nm bathochromic shifts in the absorption spectra, respectively, relative to their analogue 7. The absorption bands of compounds 11 and 12 are coincident and show bathochromic shift relative to the reference compound 7, indicating that the length of the flexible chain imposes no influence on the absorption spectra, but the flexible chain improves the performance of the electron-donating ability of thiophene. The electron donating and withdrawing groups concomitant with the degree of conjugation contribute to the effect on the spectroscopic properties and provide the means to tailor the spectroscopic properties for a given application.

### Thermal stability

**Fig. 3:**
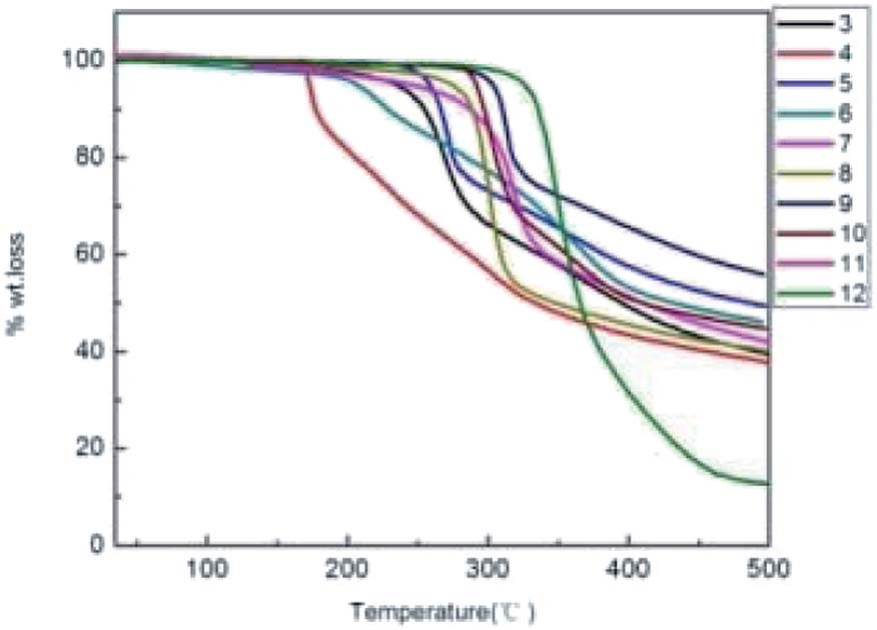
Thermogravimetric analysis of conjugated compounds 3–12 measured at a heating rate of 10 °C min^−1^ under nitrogen.

The thermal properties of the conjugated compounds 3–12 were measured by thermogravimetric analysis (TGA) under nitrogen, and the thermograms are shown in Fig. 3. All the conjugated thiophenoazomethine compounds exhibit good thermal stability except asymmetric conjugated thiophenoazomethine 4. The decomposition of symmetrical conjugated compounds 7–12 occurred slowly with an increase in temperature probably because of the greater degree of π-electron conjugation relative to asymmetrical conjugated compounds 3–6. With an increase in the π-conjugation, the thermal stability of the compounds increased. This phenomenon indicates that symmetrical conjugated compounds 7–12 are thermally more stable than asymmetrical conjugated compounds 3–6.

### Electrochemical properties

**Fig. 4:**
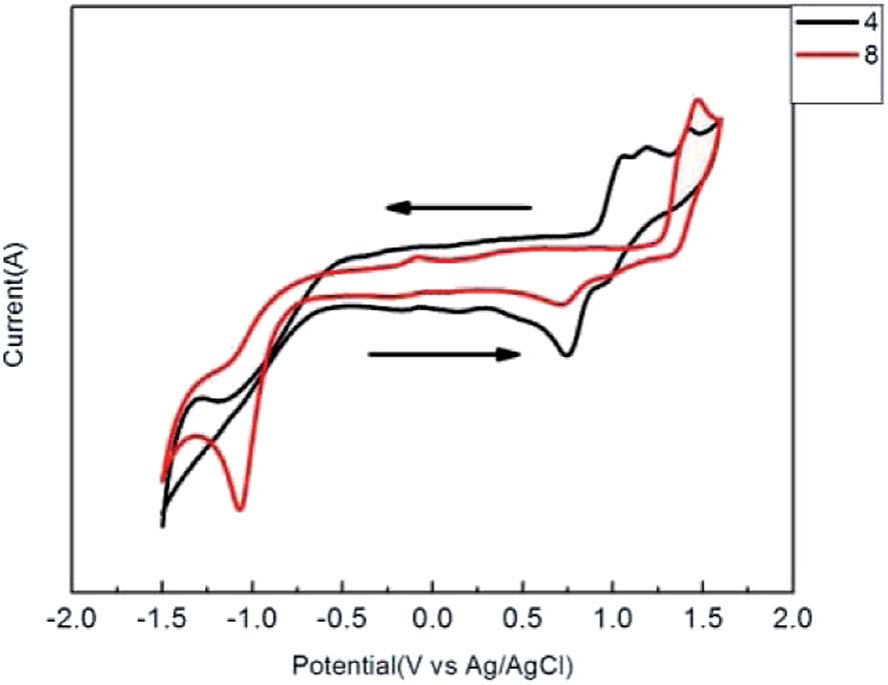
Cyclic voltammetry of conjugated thiophenoazomethines 4 and 8 at (1.0 × 10^−4^ M) concentration in dry dichloromethane.

Cyclic voltammetry (CV) experiments were performed on solutions of compounds 3–12 in deoxygenated dichloromethane with tetrabutylammonium hexafluorophosphate (Bu_4_NPF_6_) as the supporting electrolyte, an Ag/AgCl reference electrode, and platinum wire as the counter electrode. The CV plots of conjugated thiophenoazomethine compounds are shown in Fig. 4 and the corresponding data are reported in Table 1.

**Table 1:** Electrochemical properties of conjugated compounds 3–12

Compound	*λ*_onset_, (nm)	*E* ^onset^_oxid_, (V)	HOMO^a^, (eV)	*E* ^onset^_red_, (V)	LUMO^b^, (eV)	*E*_g_ c, (eV)
3	485	0.88	−5.28	−0.75	−3.65	1.63
4	505	0.90	−5.30	−0.59	−3.81	1.49
5	489	0.76	−5.16	−0.80	−3.60	1.56
6	595	1.01	−5.41	−0.54	−3.86	1.55
7	551	1.18	−5.58	−0.68	−3.72	1.86
8	560	1.22	−5.62	−0.64	−3.76	1.86
9	580	0.90	−5.30	−0.83	−3.57	1.73
10	590	1.30	−5.70	−0.33	−4.07	1.63
11	565	1.20	−5.60	−0.61	−3.79	1.81
12	565	1.20	−5.60	−0.61	−3.79	1.81

a HOMO = −(*E*^onset^_oxid_ + 4.40).

b LUMO = −(*E*^onset^_red_ + 4.40).

c *E*_g_ = LUMO − HOMO.

From the electrochemical measurements, the conjugated thiophenoazomethine compounds were found to undergo one-electron oxidation. Even though the compounds illustrated in Scheme 1 possess more than one thiophene units, and which thiophene is oxidized cannot be clearly specified. Unlike their homologous aryl azomethines,^20–22^ the formation of the thiophenoazomethine radical cation is reversible. Moreover, the oxidation potentials of thiophenoazomethines are lower than those of aryl azomethines. These desired properties are beneficial to their utility as function materials. The redox properties of conjugated thiophenoazomethine compounds can be tailored by the degree of conjugation and the use of electronic groups. For example, the terminal electron-deficient group –Br contributes to the increase in oxidation potential of compound 4 to 900 mV relative to that of ref. 3. When replaced by –NO_2_, the oxidation potential increases up to 1001 mV. In contrast, when replaced by electron-donating group –CH_3_, the oxidation potential decreases to 760 mV. When the degree of conjugation increased, the symmetric conjugated thiophenoazomethines 7–10 showed the same trend as asymmetric compounds. However, the oxidation potential increased as compared to the trend observed in asymmetric conjugated thiophenoazomethines.

The compounds 3–12 undergo not only an oxidation process, but also an irreversible one-electron reduction. The reduction process of all the conjugated thiophenoazomethines is attributed to a radical anion located on the thiophene moiety. The influence of the reduction potential by the electron groups is significant and described in Table 1.

The HOMO and LUMO energy levels were determined from the oxidation and reduction potentials,^29^ respectively. The calculated HOMO and LUMO energy levels are reported in Table 1. From the calculated values, we can see that with an increase in the degree of conjugation the HOMO level increased. However, the LUMO energy level increased only slightly. Furthermore, the electron withdrawing groups have greater extent of influence on the LUMO level than the HOMO level. The electrochemical data indicate that the degree of conjugation and terminal groups affect the oxidation and reduction potentials. Moreover, the data also imply that the compounds are stable under ambient conditions given that their HOMO energy level is below −5.5 eV. This is very important for devices with air stability.

## Conclusion

In summary, we synthesized symmetrical and asymmetrical conjugated thiophenoazomethines by employing the condensation reaction between amines and aldehydes. Because of the existence of terminal groups and the increasing degree of the conjugation, the symmetrical compounds exhibit a red-shift in the absorption spectra bands and lower HOMO energy levels compared with those of asymmetrical compounds. The terminal withdrawing groups –Br and –NO_2_ and donating groups –NH_2_ and –CH_3_ form electronic push–pull, push–push and pull–pull systems, which cause the change in HOMO–LUMO energy gap. Thus, we can utilize the terminal groups and conjugation effect to adjust the photophysical and electrochemical properties for designing the compounds. All the symmetrical conjugated thiophenoazomethines demonstrated excellent thermal stability and lower HOMO energy levels, making them promising candidates for organic devices. Further, we will utilize the azomethines to synthesize new conjugated compounds and investigate their applications in devices. The investigation is ongoing and will be reported in future.

## Conflicts of interest

There are no conflicts to declare.

## Supplementary Material

RA-008-C8RA00570B-s001
