# Supplementary material for: Tuning of the photophysical and electrochemical properties of symmetric and asymmetric conjugated thiophenoazomethines
Source: RSC Adv. 2018 Apr 3;8(23):12779–84. doi: 10.1039/c8ra00570b (PMC9079336; doi:10.1039/c8ra00570b)
Supplement: RA-008-C8RA00570B-s001 [file RA-008-C8RA00570B-s001.pdf]

Electronic Supplementary Material (ESI) for RSC Advances.
This journal is © The Royal Society of Chemistry 2018

Supporting information

Tuning of the Photophysics and Electrochemistry of Symmetric and Asymmetric conjugated Thiopheno Azomethines

Shengzhen Liu[ab], Ti Wu[b*], Qi Zhu[ab], Jialing Pu[b], Guangxue Chen[a], Weimin Zhang[b], Zhongxiao Li[b]

a. South China University of Technology, State Key Laboratory of Pulp and Paper Engineering, 381 Wushan Road, Tianhe District, Guangzhou, P.R.China, 510641
b. Beijing Institute Of Graphic Communication, Information Recording Material Lab,Lab of Printing &Packing Materials and Technology, No.1 (band -2)Xinghua Street, Daxing District, Beijing, P. R. China, 102600

1. Experimental procedures

General method

All chemicals were of reagent grade and used without further purification unless otherwise indicated. 2,5-diamino-thiophene-3,4-dicarboxylic acid diethyl ester and 2-aminothiophene-3-carbonitrile synthesized as described previously[27][28]. The ^{1}H and ^{13}C NMR spectra were recorded with a Bruker 300 MHz NMR spectrometer by using $CDCl_3$ as the solvent. Chemical shifts (δ) are given in ppm relative to $CDCl_3$(δ=7.26 ppm for ^{1}H and 77 ppm for ^{13}C). MS data were recorded with a mass spectrometer. The TGA analyses were performed with a thermal analysis system that was set to a heating rate of 10 °C min^{-1} under nitrogen at room temperature. The UV/Vis absorption spectra of the conjugated thiopheno azomethines compounds were recorded in trichloromethane with a UV/Vis spectrophotometer. Cyclic voltammograms were recorded under nitrogen using a one-compartment, three-electrode cell, equipped with a platinum wire as counter electrode, platinum plate as working electrode, saturated Ag/Ag+ as the reference electrode. The supporting electrolyte was 0.1M tetrabutylammounium hexafluorophosphate (Bu_4NPF_6) and the measurements were conducted on compounds in deoxygenated DCM.

Synthetic procedures

Synthesis of 3: ^1H NMR(300MHz, $CDCl_3$) : δ (ppm)=8.08(s, H), 7.44(d, H), 7.34(s, H), 7.07(s, H), 6.31(s, 2H), 4.44-4.38(m, 2H), 4.28-4.21(m, 2H), 1.47-1.42(t, 3H), 1.33-1.28(t, 3H); ^{13}C NMR(75MHz, CDCl3) : δ(ppm)=165.3, 164.4, 159.3, 145.9, 142.5, 133.9, 131.0, 130.1, 129.3, 127.7, 61.4, 60.2, 14.1。 MS(EI-MS): calcd. for $C_{15}H_{16}N_2O_4S_2$ $[M]^+$ 352.06, found 352.

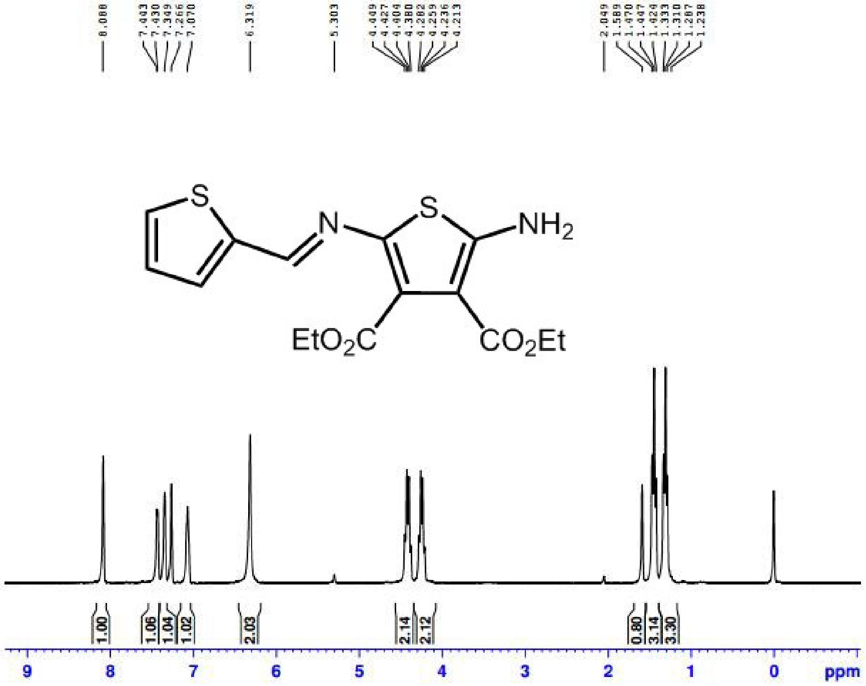

Figure S1. ^1H NMR spectrum of compound 3

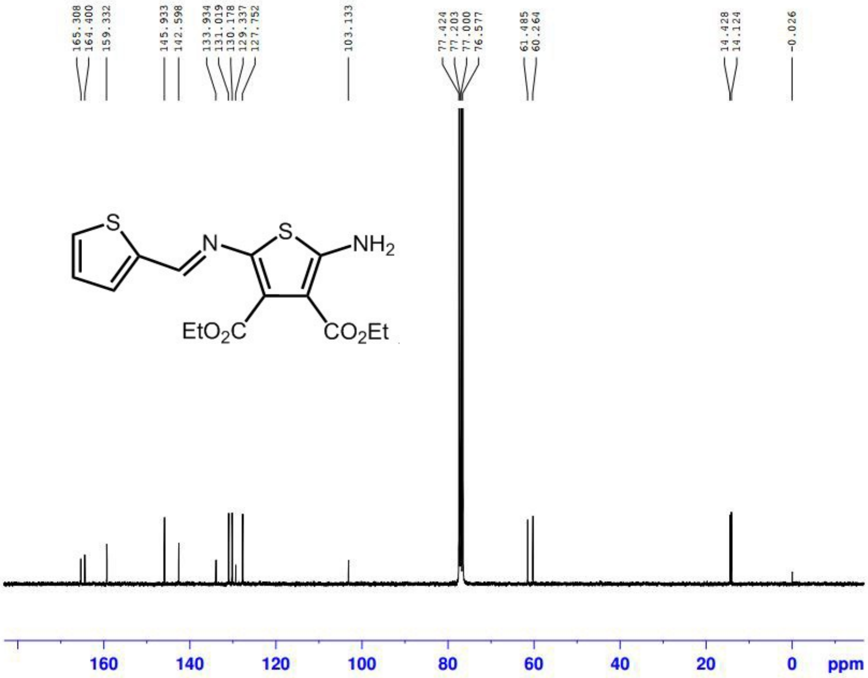

Figure S2. ^{13}C NMR spectrum of compound 3

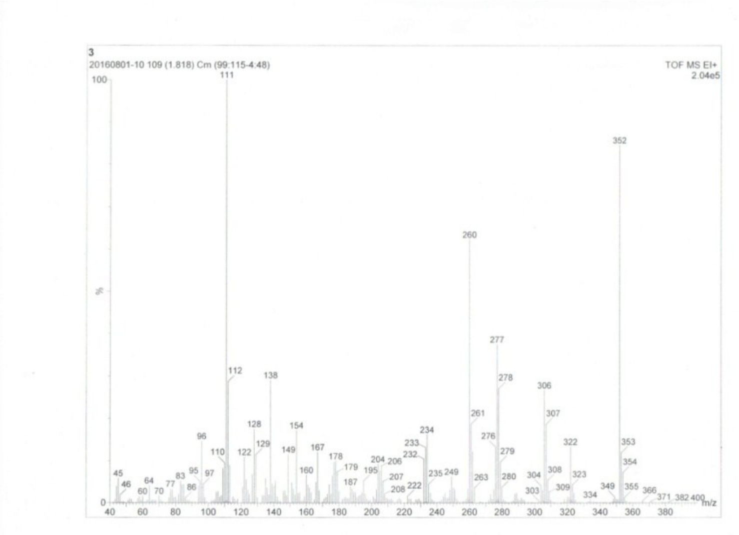

Figure S3. MS spectrum of compound 3

Synthesis of 4: In a 100 mL round bottom flask were dissolved 1(258mg, 1 mmol) in absolute isopropanol(60 mL), 5-bromothiophene-2-carboxaldehyde (287mg, 1.5mmol) and a catalytic amount of trifluoroacetic acid (TFA) was added. The mixture was refluxed for 16 hours. The solvent was removed in vacuo, and the product was purified by chromatography on a silica gel column (dichloromethane), then recrystallized with ethanol to yield 4 (194mg, 45 % yield) as a red solid. ^{1}H NMR(300MHz，CDCl3)： δ (ppm)=7.93(s, H), 7.11-7.02(m, 2H), 6.33(s, 2H), 4.44-4.37(m, 2H), 4.28-4.21(m, 2H), 1.47-1.42(t, 3H), 1.33-1.29(t,3H); ^{13}C NMR(75MHz, CDCl3)：δ(ppm)=165.1, 164.3, 159.5, 144.6, 144.1, 133.3, 130.8, 130.7, 129.9, 118.3, 103.1, 61.5, 60.3, 14.4, 14.1. MS(EI-MS): calcd. for $C_{15}H_{15}BrN_2O_4S_2$ $[M]^+$ 431.3, found 432.

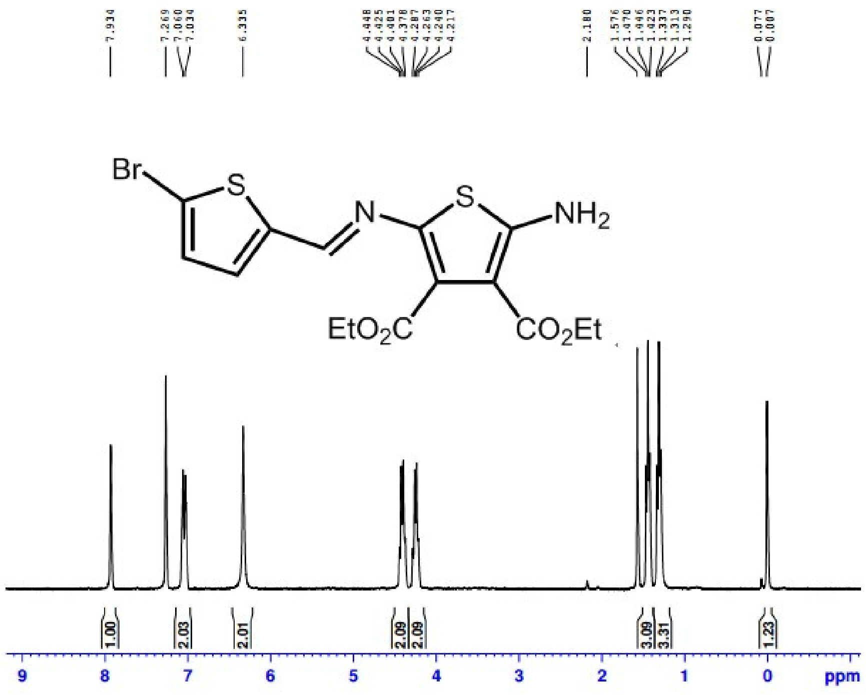

Figure S4. ^{1}H NMR spectrum of compound 4

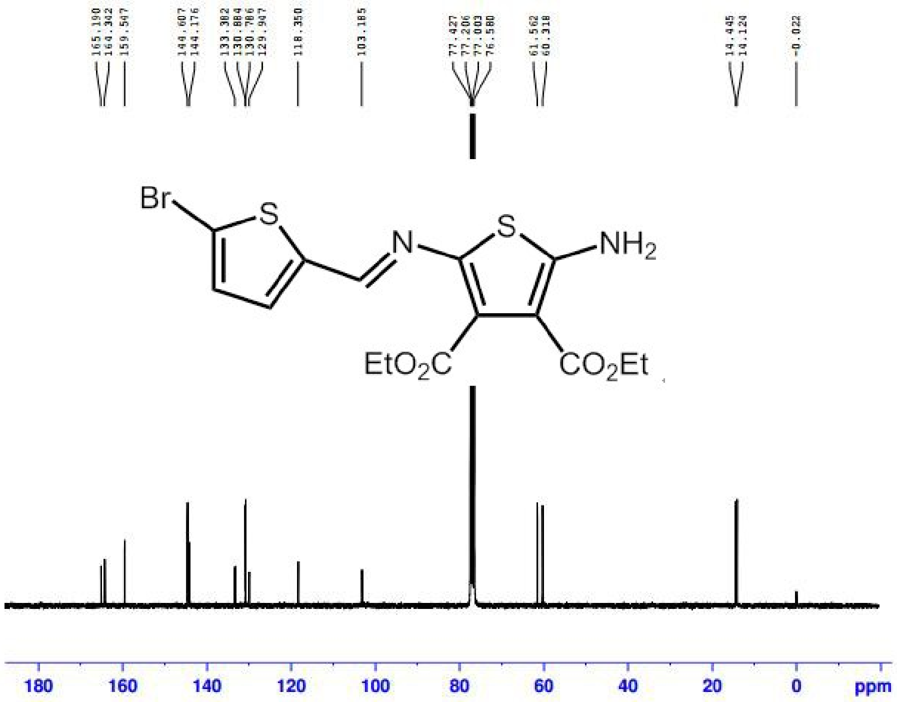

Figure S5. ^{13}C NMR spectrum of compound 4

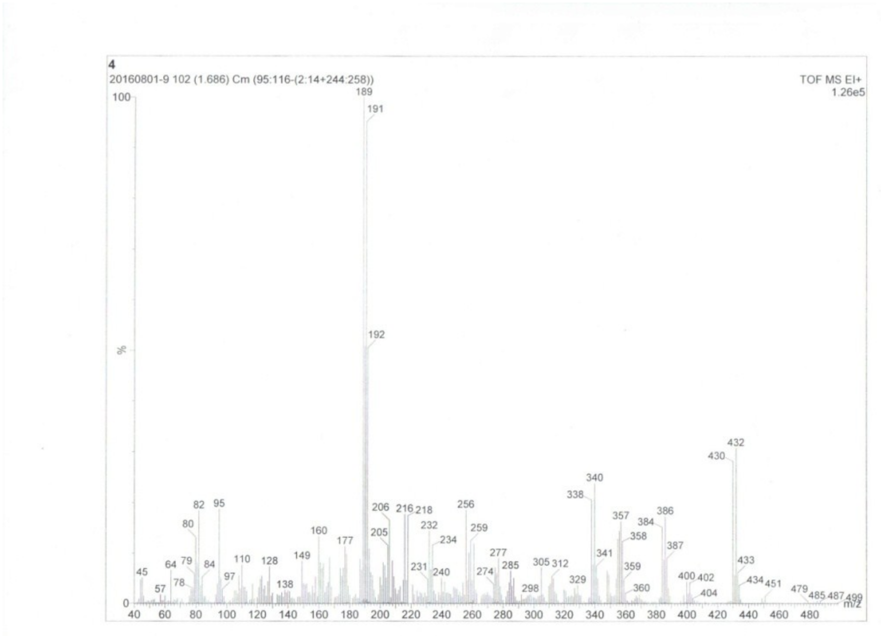

Figure S6. MS spectrum of compound 4

Synthesis of 5: The synthesis method was same as compound 4. ^{1}H NMR(300MHz， CDCl3)： δ (ppm)=7.99(s, H), 7.16(s, H), 6.73(s, H), 6.27(s, 2H), 4.44-4.37(m, 2H), 4.28-4.21(m, 2H), 2.52(s, 3H), 1.47-1.42(t, 3H), 1.33-1.28(t,3H); ^{13}C NMR(75MHz, CDCl3)： δ(ppm)=165.4, 164.4, 146.2, 145.9, 140.4, 134.4, 131.7, 128.4, 126.3, 102.9, 61.4, 60.2, 14.4, 14.1. MS(EI-MS): calcd. for $C_{16}H_{18}N_2O_4S_2$ $[M]^+$ 366, found 366.

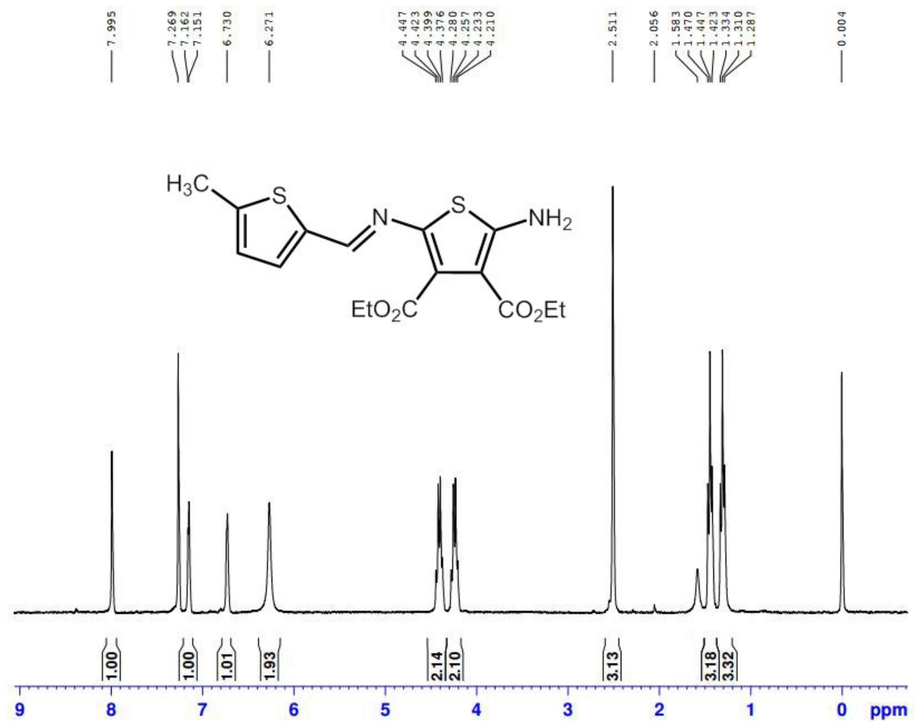

Figure S7. ^{1}H NMR spectrum of compound 5

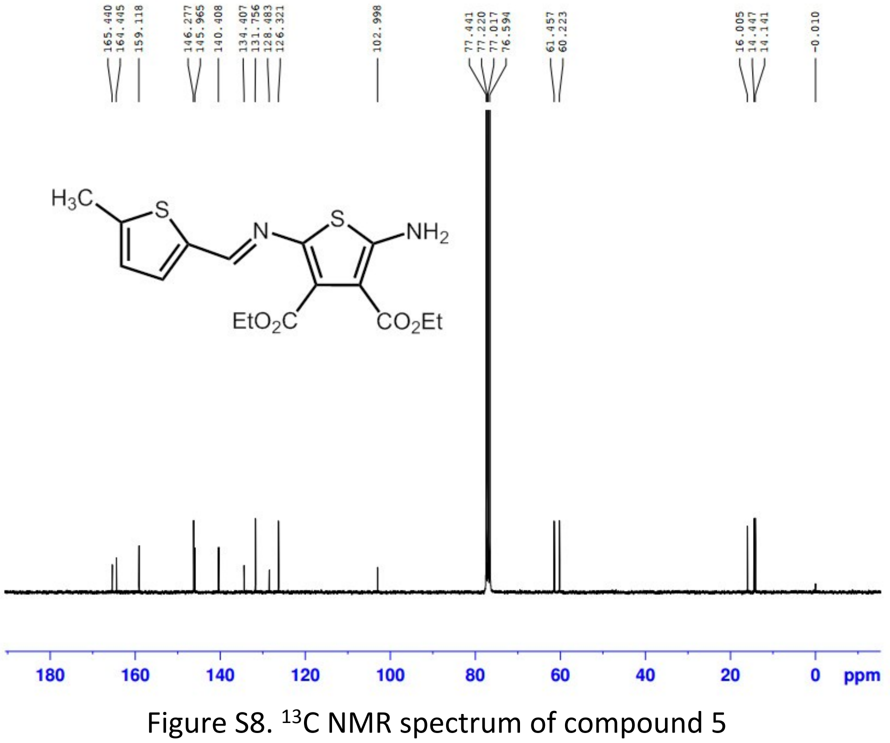

Figure S8. ^{13}C NMR spectrum of compound 5

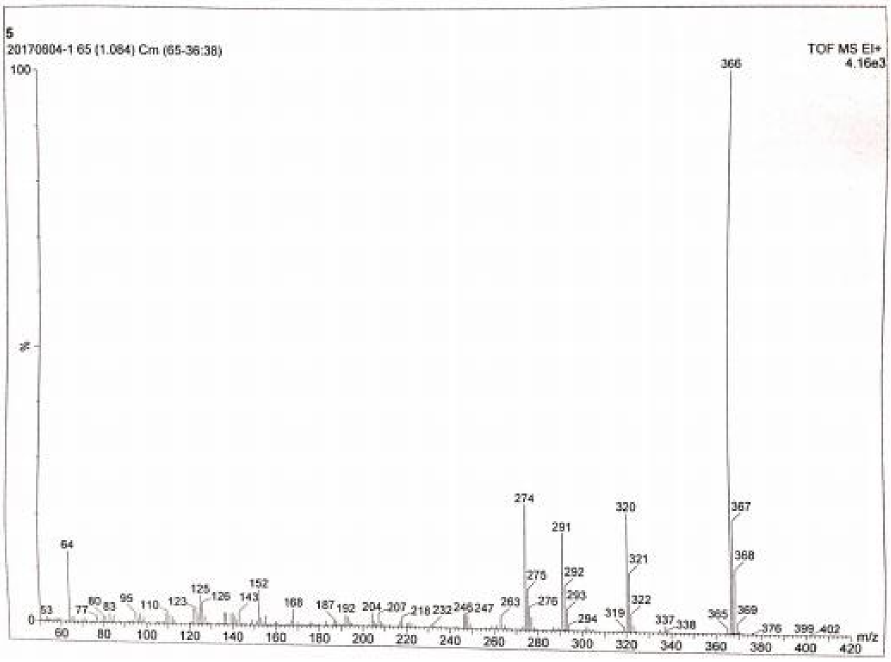

Figure S9. MS spectrum of compound 5

Synthesis of 6: The synthesis method was same as compound 4. ^{1}H NMR(300MHz，CDCl3)：δ (ppm)=7.93(s, H), 7.85(s, H), 7.21(s, H), 6.54(s, 2H), 4.47-4.40(m, 2H), 4.29-4.22(m, 2H), 1.48-1.43(t, 3H), 1.34-1.29(t,3H); ^{13}C NMR(75MHz, CDCl3)：δ(ppm)=164.7, 164.1, 160.9, 153.0, 148.7, 142.6, 133.3, 131.8, 128.8, 128.0, 103.7, 61.9, 60.6, 14.4, 14.1. MS(EI-MS): calcd. for $C_{15}H_{15}N_3O_6S_2$ $[M]^+$ 397, found 397.

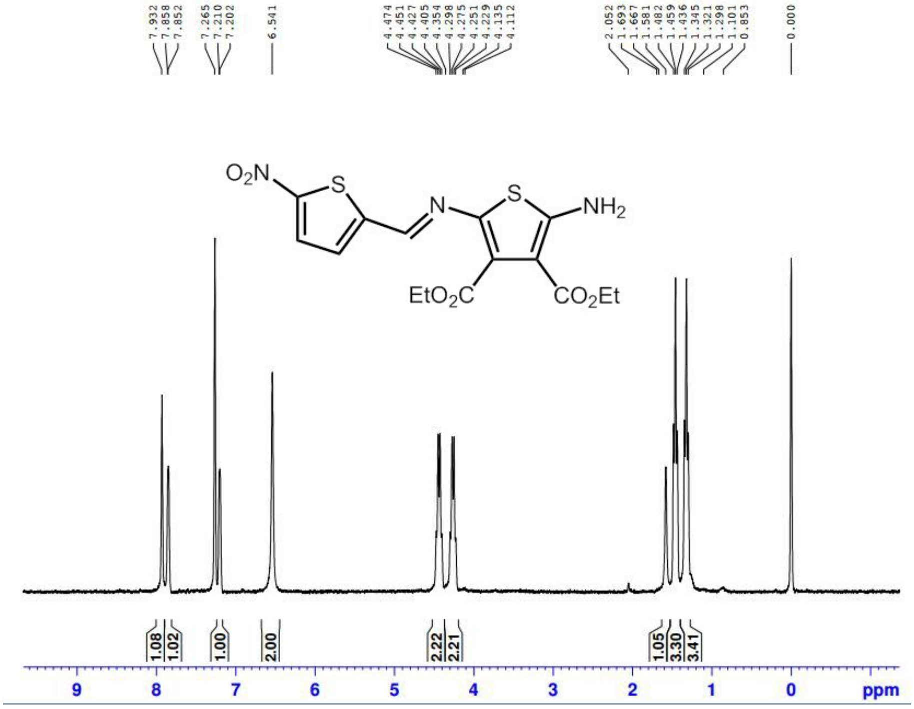

Figure S10. ^{1}H NMR spectrum of compound 6

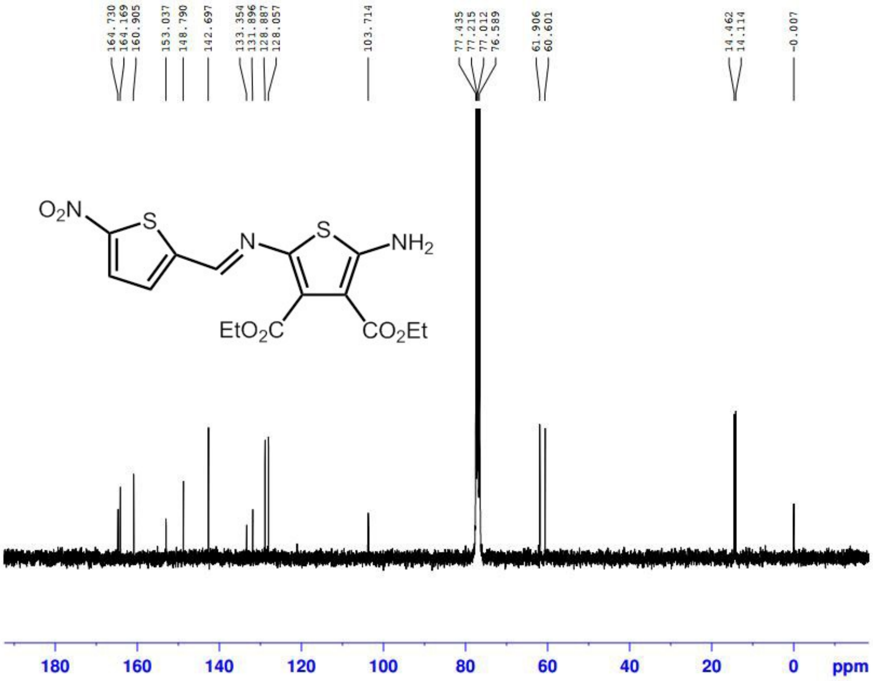

Figure S11. ^{13}C NMR spectrum of compound 6

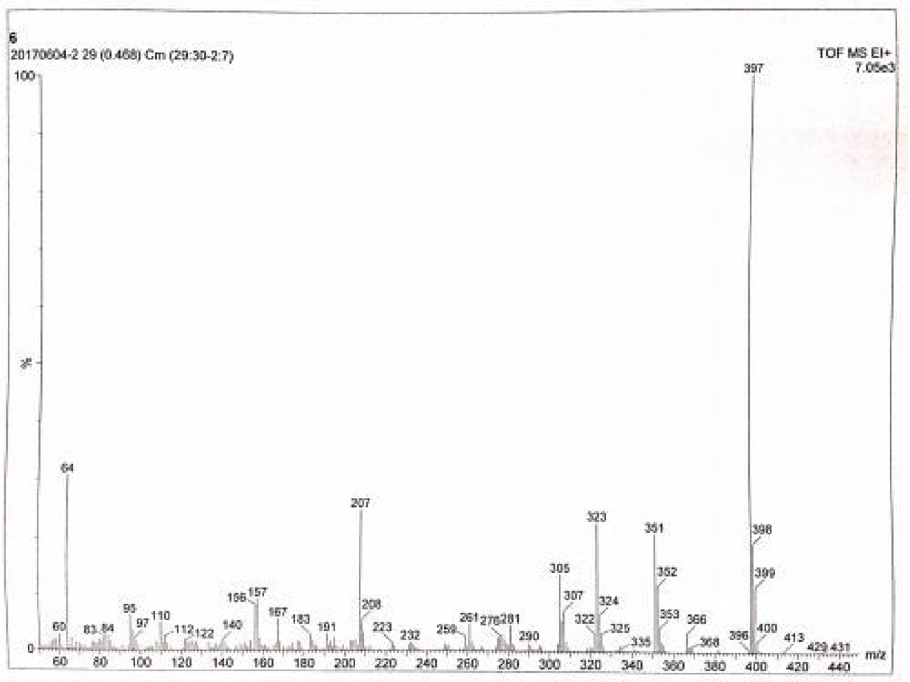

Figure S12. MS spectrum of compound 6

Synthesis of 7: ^{1}H NMR(300MHz，CDCl3)： δ (ppm)=8.50(s,2H), 7.59-7.52(m, 4H), 7.14(s, 2H), 4.43-4.36(t, 4H), 1.47-1.33(t, 6H); ^{13}C NMR(75MHz,CDCl3)：δ(ppm)=163.2, 152.1, 148.9, 142.0, 133.4, 132.3, 128.1, 127.0, 61.3, 14.2. MS(EI-MS): calcd. for $C_{20}H_{18}N_2O_4S_3$ $[M]^+$ 446.5, found 446.

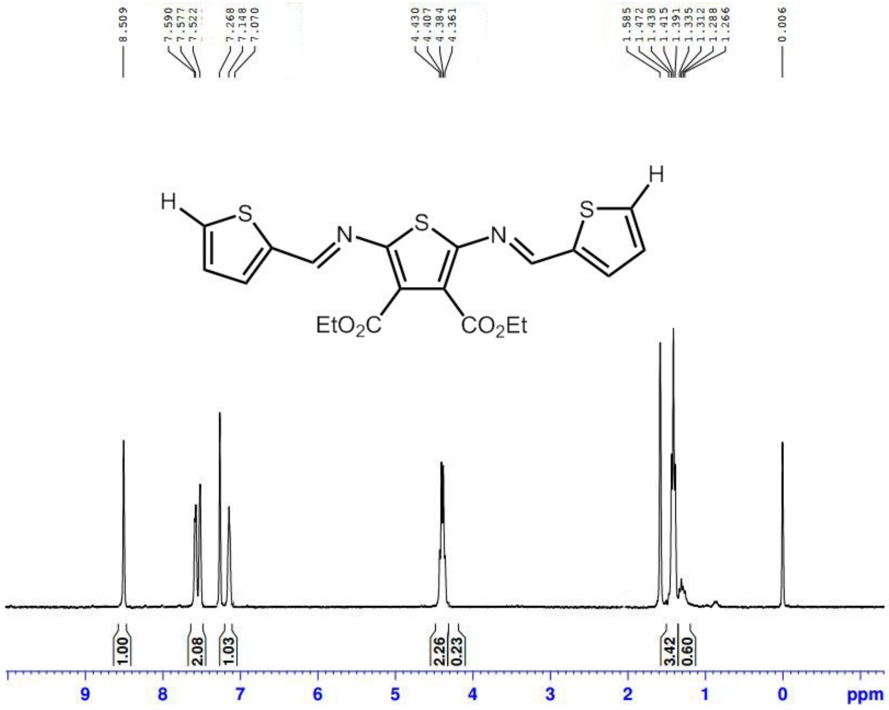

Figure S13. ^{1}H NMR spectrum of compound 7

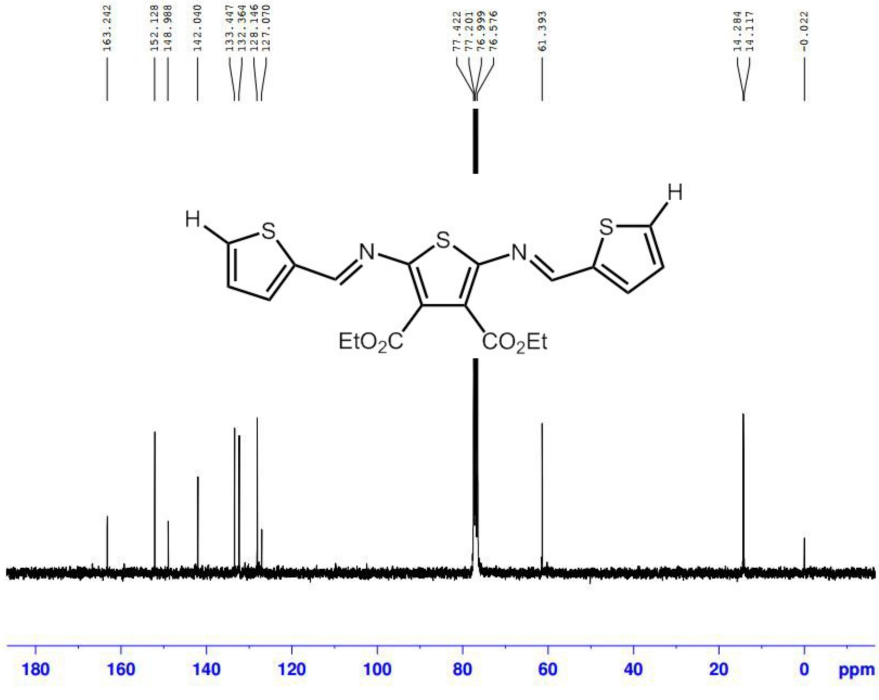

Figure S14. ^{13}C NMR spectrum of compound 7

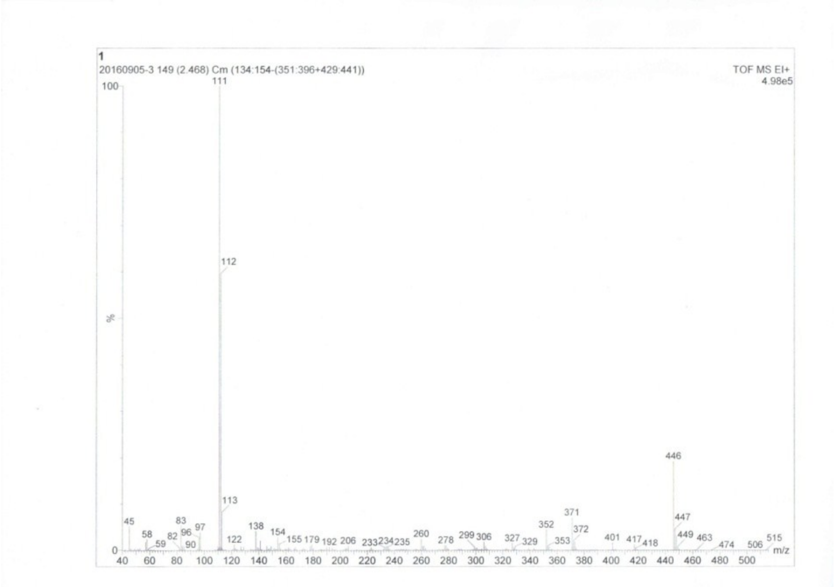

Figure S15. MS spectrum of compound 7

Synthesis of 8: 5-bromothiophene-2-carboxaldehyde(96mg, 0.5mmol) was dissolved in anhydrous toluene(30ml) at 0oC with DABCO (58 mg, 0.5 mmol) and the slow addition of TiCl4(1.0 M solution in toluene) (510 μL, 0.5 mmol).then 1(64.5 mg, 0.25 mmol) was added and the mixture was then refluxed overnight. The solvent was removed in vacuo, and the product was purified by chromatography on a silica gel column (dichloromethane), then recrystallized with ethanol to yield 8 (60mg, 40% yield) as a red solid. ^{1}H NMR(300MHz， CDCl3)： δ (ppm)=8.31(s, 2H), 7.26-7.23(t, 2H), 7.10-7.08(d, 2H), 4.42-4.37(t, 4H), 1.43-1.38(t, 6H); ^{13}C NMR(75MHz,CDCl3)： δ(ppm)=163.0, 150.9, 148.6, 143.5, 133.5, 131.2, 127.6, 121.1, 61.4, 14.3. MS(MALDI-TOF): calcd. for $C_{20}H_{16}Br_2N_2O_4S_3$ $[M]^+$604.3, found 604.

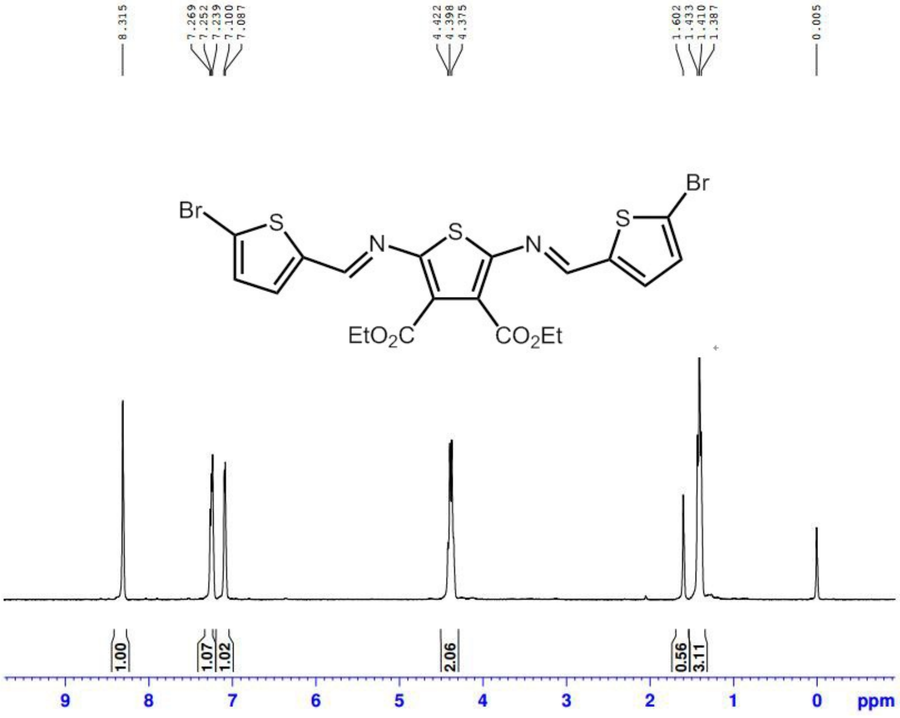

Figure S16. ^{1}H NMR spectrum of compound 8

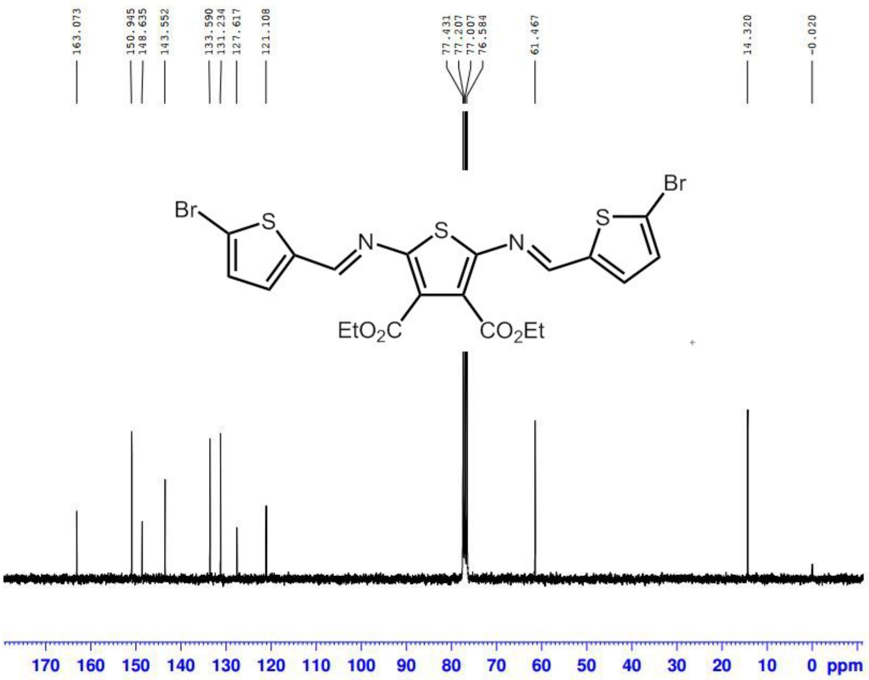

Figure S17. ^{13}C NMR spectrum of compound 8

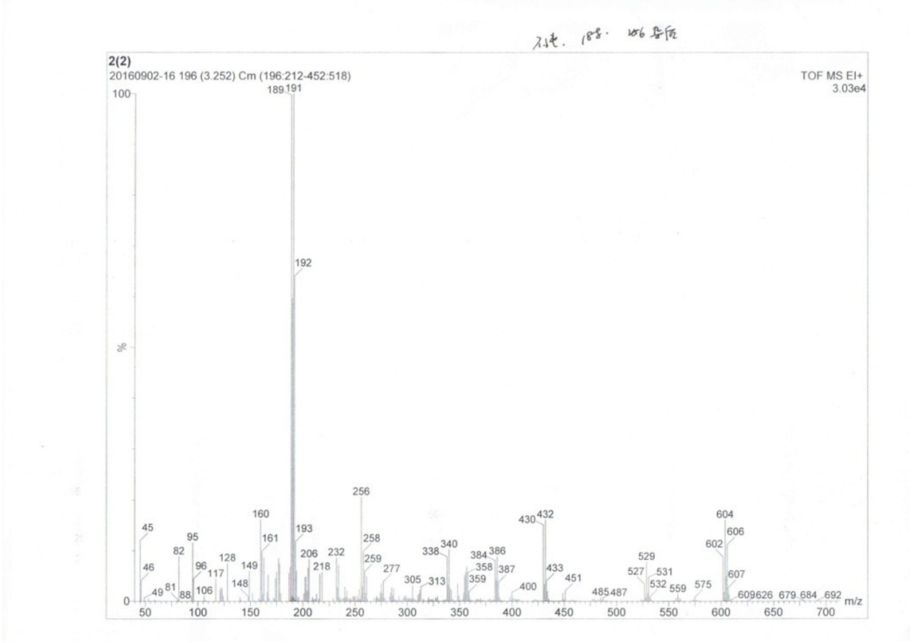

Figure S18. MS spectrum of compound 8

Synthesis of 9: The synthesis method was same as compound 8. ^{1}H NMR(300MHz， CDCl3)：δ (ppm)=8.38(s, 2H), 7.31-7.26(d, 2H), 6.80(s, 2H), 4.41-4.34(t, 4H), 2.58-2.50(t, 6H), 1.43-1.38(t, 6H); ^{13}C NMR(75MHz,CDCl3)： δ(ppm)=163.3, 151.9, 149.0, 148.4, 139.9, 134.1, 126.8, 126.1, 61.2, 16.1, 14.3. MS(EI-MS): calcd. for $C_{22}H_{22}N_2O_4S_3$ [M+H$^+$]474, found 474.

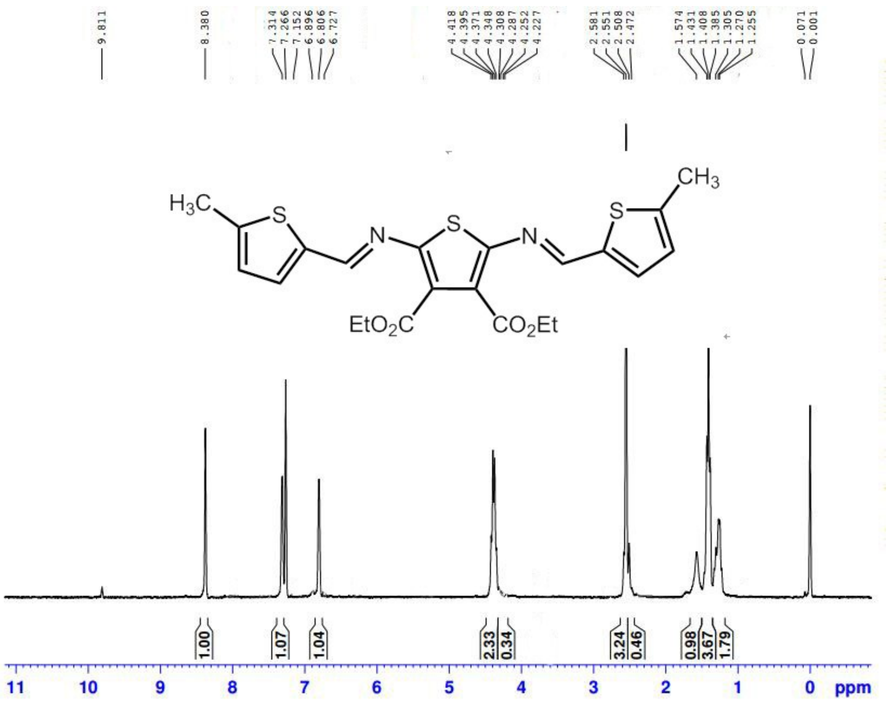

Figure S19. ^{1}H NMR spectrum of compound 9

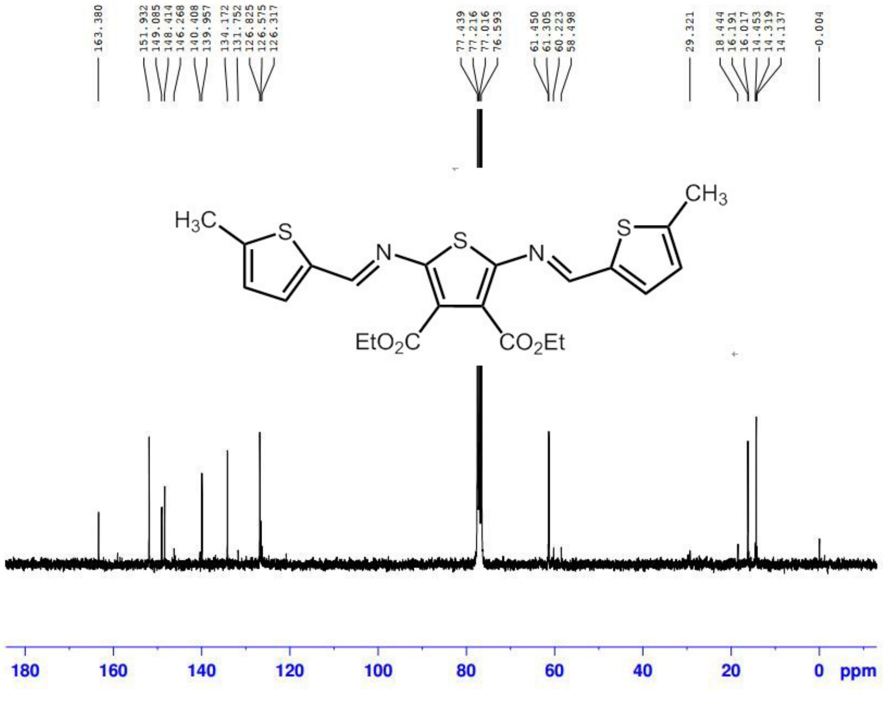

Figure S20. ^{13}C NMR spectrum of compound 9

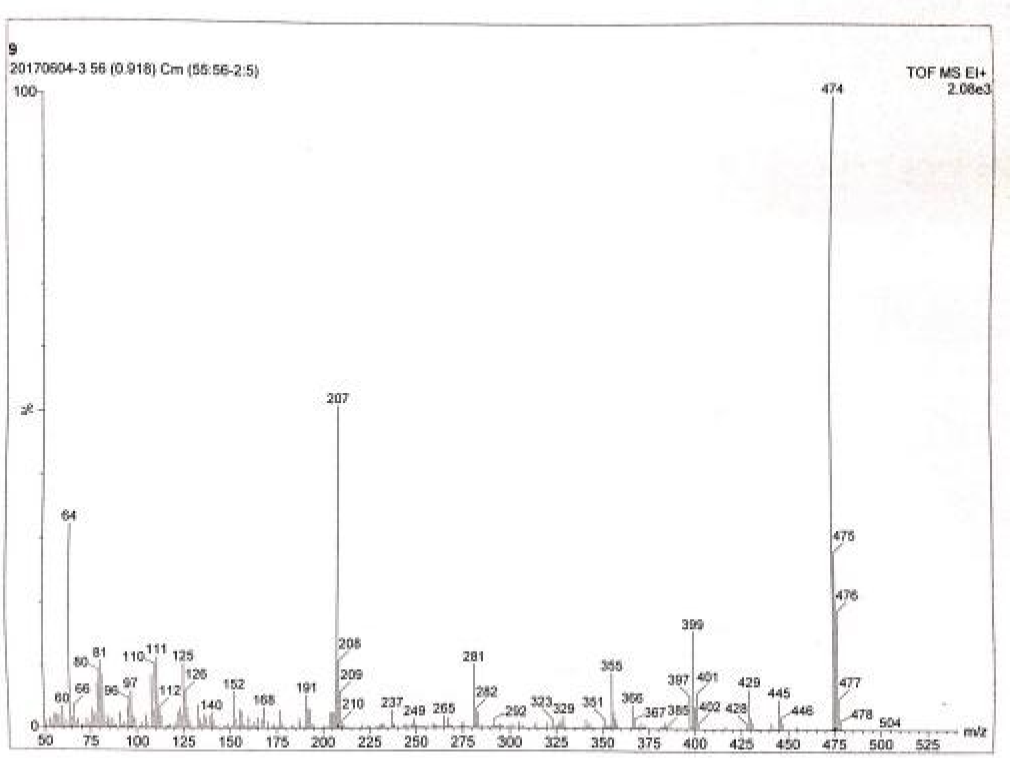

Figure S21. MS spectrum of compound 9

Synthesis of 10: The synthesis method was same as compound 8. ^{1}H NMR(300MHz， CDCl3)：δ (ppm)=8.45(s, 2H), 8.10-7.91(d, 2H), 7.43(s, 2H), 4.44-4.41(t, 4H), 1.30-1.23(t, 6H); ^{13}C NMR(75MHz,CDCl3)：δ(ppm)=155.1, 150.9, 148.3, 146.9, 131.0, 128.5, 61.9, 14.3. MS(EI-MS): calcd. for $C_{20}H_{16}N_4O_8S_3$ $[M]^+$ 536, found 536.

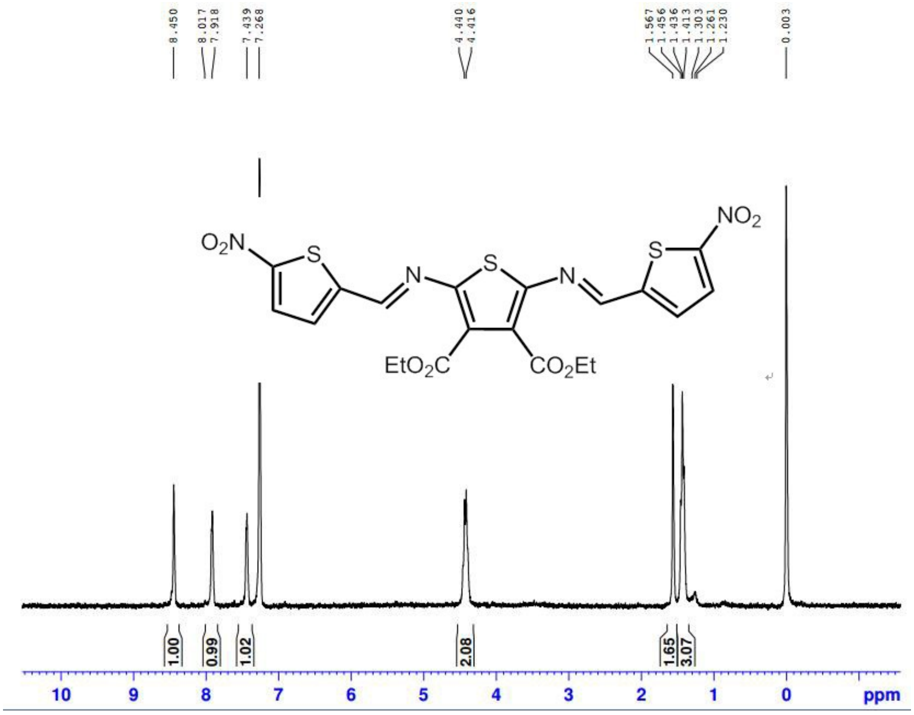

Figure S22. ^{1}H NMR spectrum of compound 10

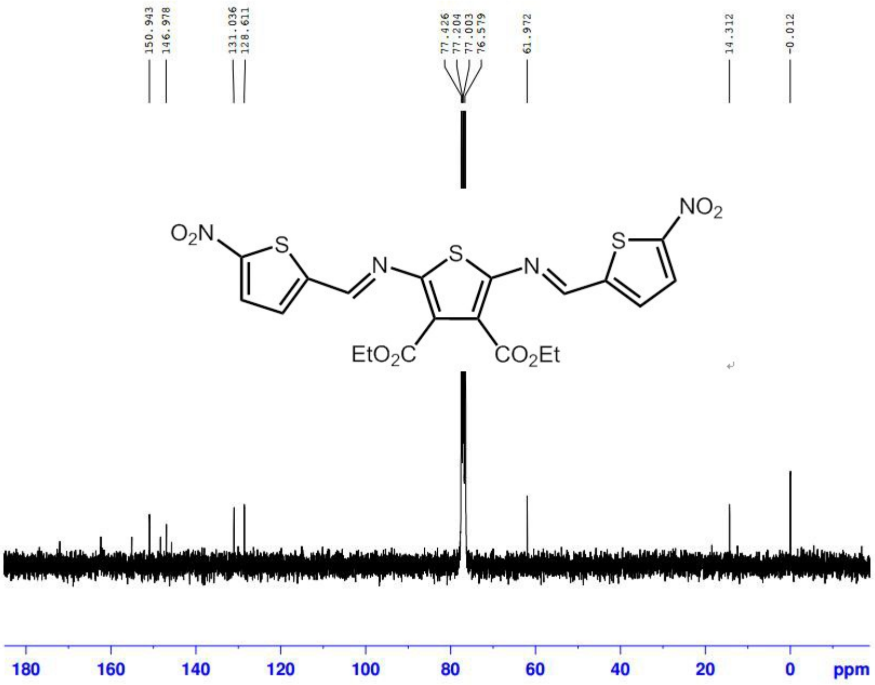

Figure S23. ^{13}C NMR spectrum of compound 10

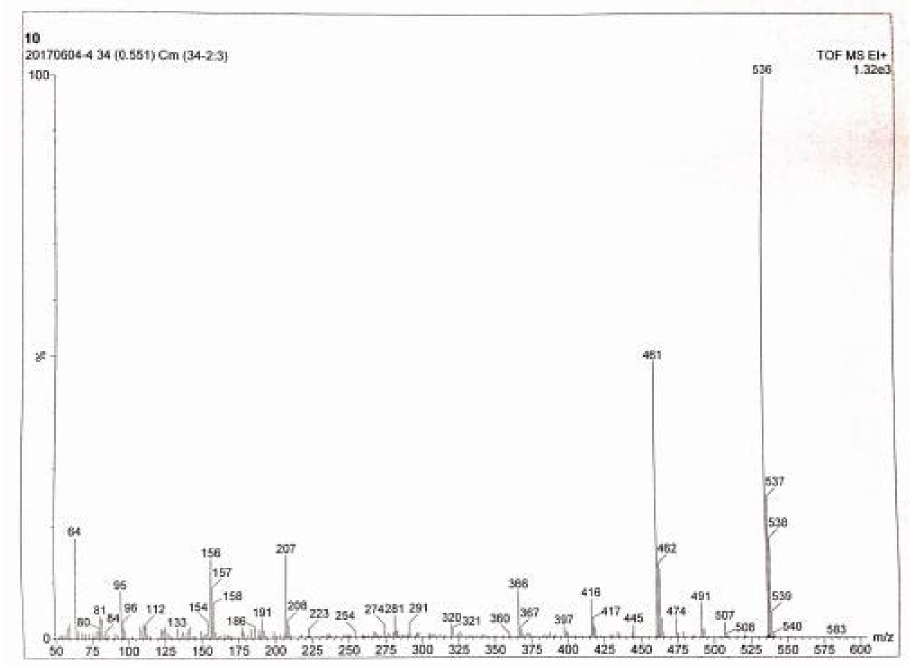

Figure S24. MS spectrum of compound 10

Synthesis of 3,4-dihexylthiophene: 3,4-dibromothiophene(4.89g， 20mmol) was dissolved in anhydrous tetrahydrofuran(150ml) under nitrogen, 1,3-Bis(diphenylphosphino)propane nickel(II) chloride(0.2g, 0.4mmol) was added and the slow addition of hexylmagnesium bromide (1.0 M solution in tetrahydrofuran) (67mL, 67 mmol), the mixture was then refluxed for 18 hours. Cooling to room temperature, then filtrate, the solution was washed with aqueous HCl (10% w/w). The organic phase was washed with water, dried with MgSO4, filtered and the solvent was evaporated. The product was purified by chromatography on a silica gel column (Petroleum ether) to yield 3,4-dihexylthiophene(2.95g, 58% yield) as colorless oil. ^{1}H NMR(300MHz， CDCl3)： δ (ppm)= 6.98(s,2H), 2.72-2.55(m, 4H), 1.69(s, 4H), 1.39(s, 12H), 0.98(s, 6H); ^{13}C NMR(75MHz, CDCl3)：δ(ppm)=143.2, 125.0, 31.7, 31.4, 31.0, 30.3, 29.9, 29.8, 29.7, 29.3, 22.5, 22.3, 14.1, 14.0. MS(EI-MS): calcd. for $C_{16}H_{28}S$ $[M]^+$ 252.4, found 252.

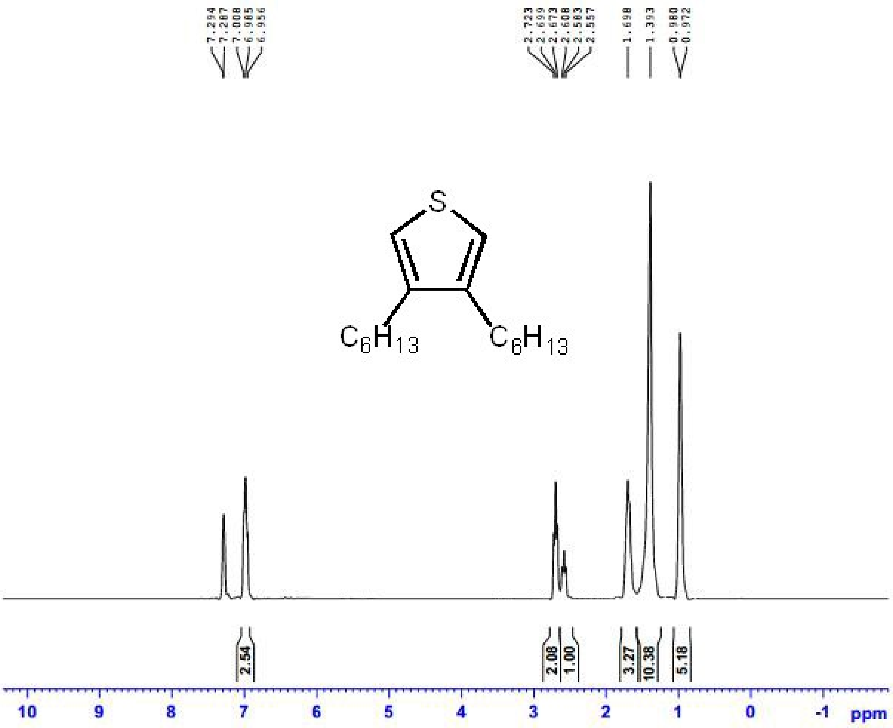

Figure S25. ^{1}H NMR spectrum of compound 3,4-dihexylthiophene

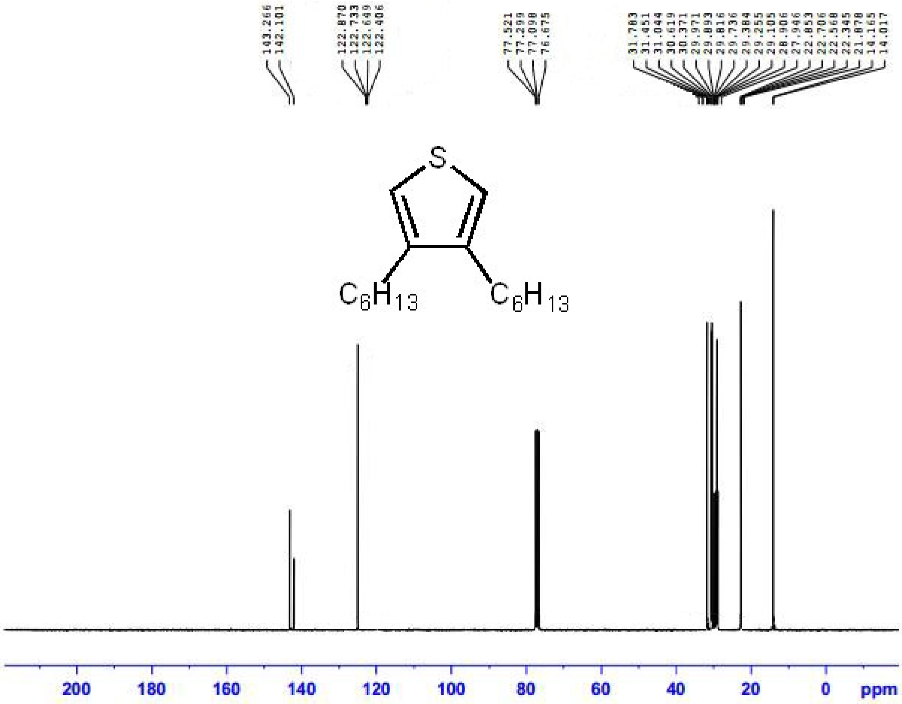

Figure S26. ^{13}C NMR spectrum of compound 3,4-dihexylthiophene

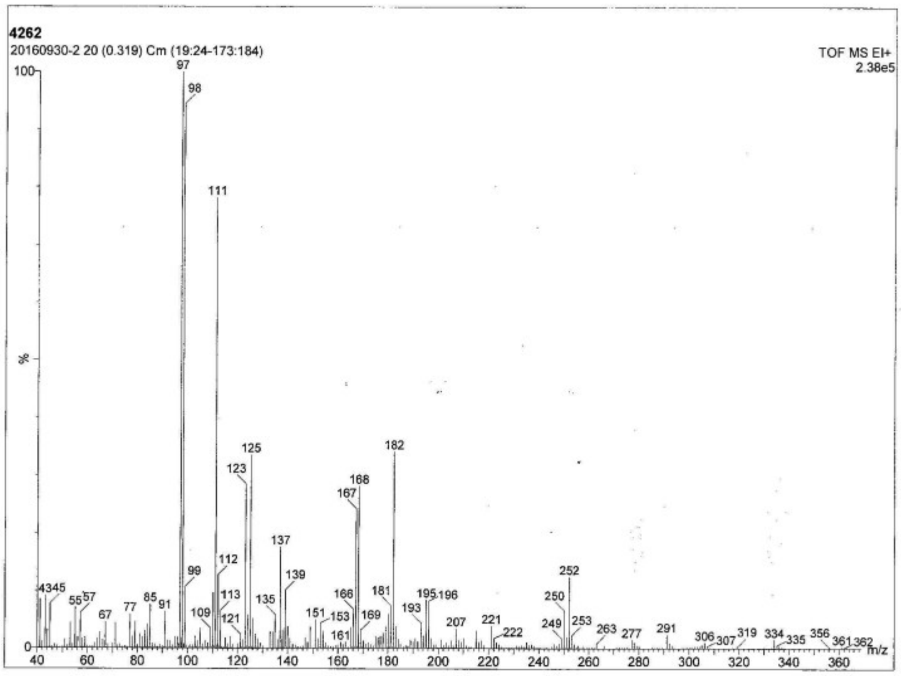

Figure S27. MS spectrum of compound 3,4-dihexylthiophene

Synthesis of 3,4-dihexylthiophene-2,5-dicarbaldehyde: 3,4-dihexylthiophene (2.32g, 9.24mmol) and freshly distilled TMEDA(4ml, 24mmol) dissolved in anhydrous hexanes(80ml) under nitrogen. n-BuLi(1.6 M in hexane, 15mL, 24mmol) was added drop-wise, reflux for 1.5 h, THF (40 mL) was added and the solution was cooled to -50 °C. Absolute anhydrous DMF (4mL, 54 mmol) was added drop-wise. Heating to room temperature and stirred for 2.5h, the reaction mixture was hydrolyzed with water (100 mL) and extracted with ether. dried with MgSO4, filtered and the solvent was evaporated. The product was purified by chromatography on a silica gel column (Petroleum ether/dichloromethane,1:1) to yield 3,4-dihexylthiophene -2,5-dicarbaldehyde (1.12g, 40% yield) as colorless oil. ^{1}H NMR(300MHz，CDCl3)：δ(ppm)=10.11(s, 2H), 2.92-2.87(t, 4H), 1.63-1.53(m, 4H), 1.43-1.32(m, 12H), 0.91-0.87(t, 6H); ^{13}C NMR(75MHz, CDCl3)：δ(ppm)=183.3, 152.4, 151.7, 143.2, 32.1, 31.4, 29.5, 29.2, 27.4, 26.9, 26.6, 22.5, 14.0. MS(EI-MS): calcd. for $C_{18}H_{28}O_2S$ $[M]^+$ 308.4, found 308.

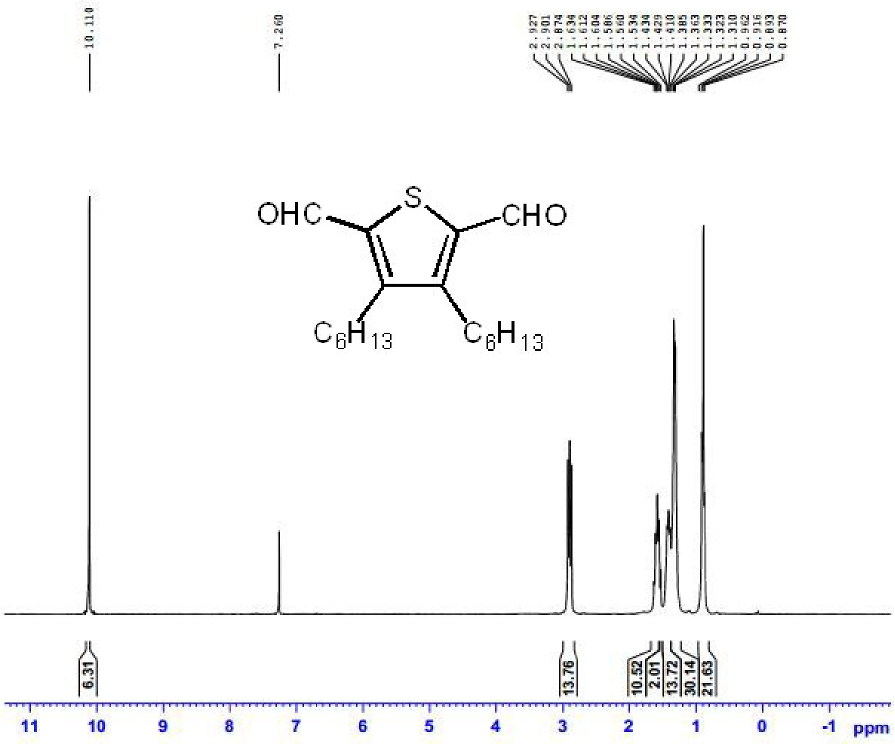

Figure S28. ^{1}H NMR spectrum of compound 3,4-dihexylthiophene -2,5-dicarbaldehyde

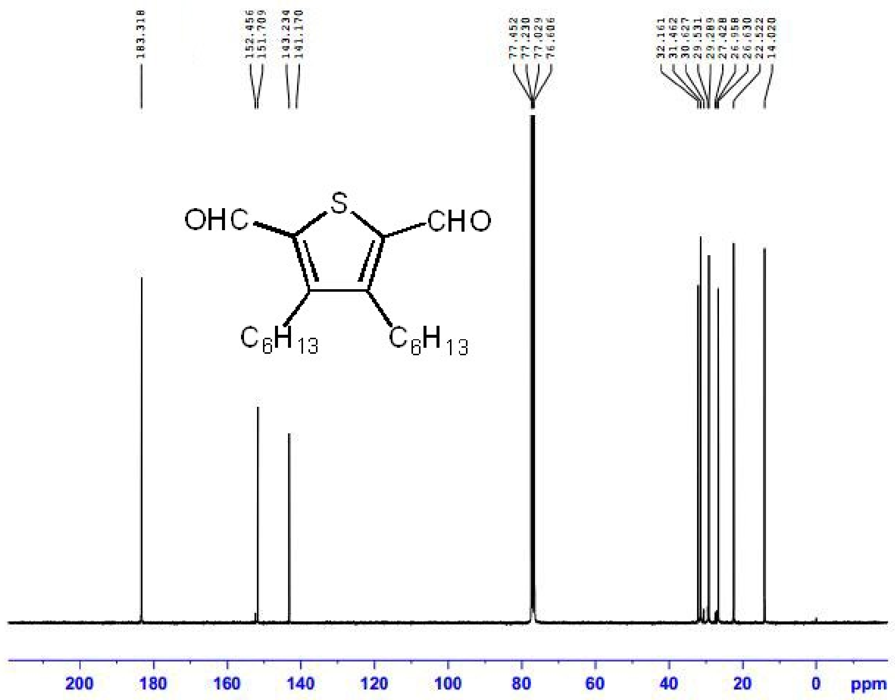

Figure S29. ^{13}C NMR spectrum of compound 3,4-dihexylthiophene -2,5-dicarbaldehyde

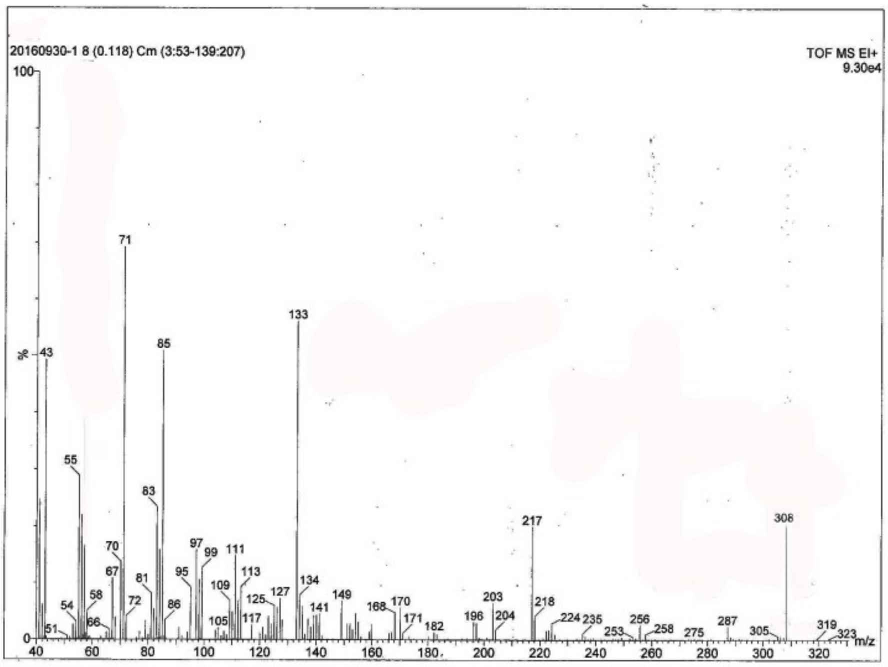

Figure S30. MS spectrum of compound 3,4-dihexylthiophene -2,5-dicarbaldehyde

Synthesis of 11: In a 100 mL round bottom flask were dissolved 2(132.68mg, 1.07 mmol) in butyl alcohol (20 mL), 3,4-dihexylthiophene -2,5-dicarbaldehyde (64.7mg, 0.21mmol) was added. The mixture was refluxed for 48 hours. The solvent was removed in vacuo, and the product was purified by chromatography on a silica gel column (dichloromethane), then recrystallized with ethanol to yield 11 (84mg, 77 % yield) as a red solid. ^{1}H NMR(300MHz，CDCl3)：δ(ppm)=8.69(s, 2H), 7.15-7.10(m, 4H), 2.87-2.84(d, 4H), 1.57(s, 4H), 1.43-1.26(m, 12H), 0.89(s, 6H); ^{13}C NMR(75MHz, CDCl3)：δ(ppm)=162.9, 152.3, 150.1, 140.8, 128.2, 121.9, 114.5，104.7, 31.7, 31.5, 29.3, 27.2, 22.5, 14.0. MS(MALDI-TOF) calcd. for $C_{18}H_{32}N_4S_3$ $[M]^+$ 520.7, found 521.1

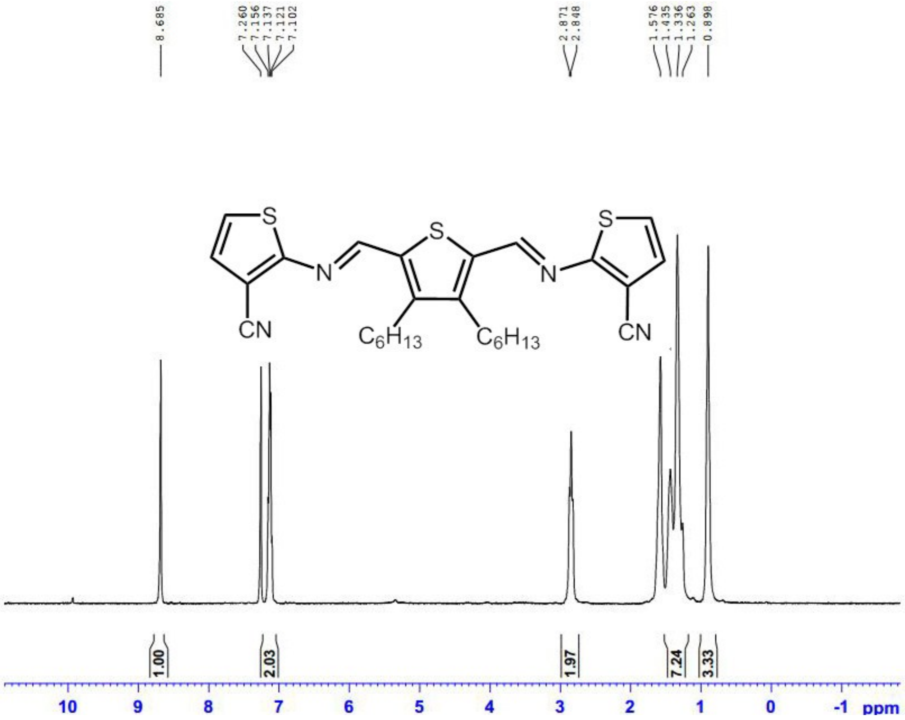

Figure S31. ^{1}H NMR spectrum of compound 11

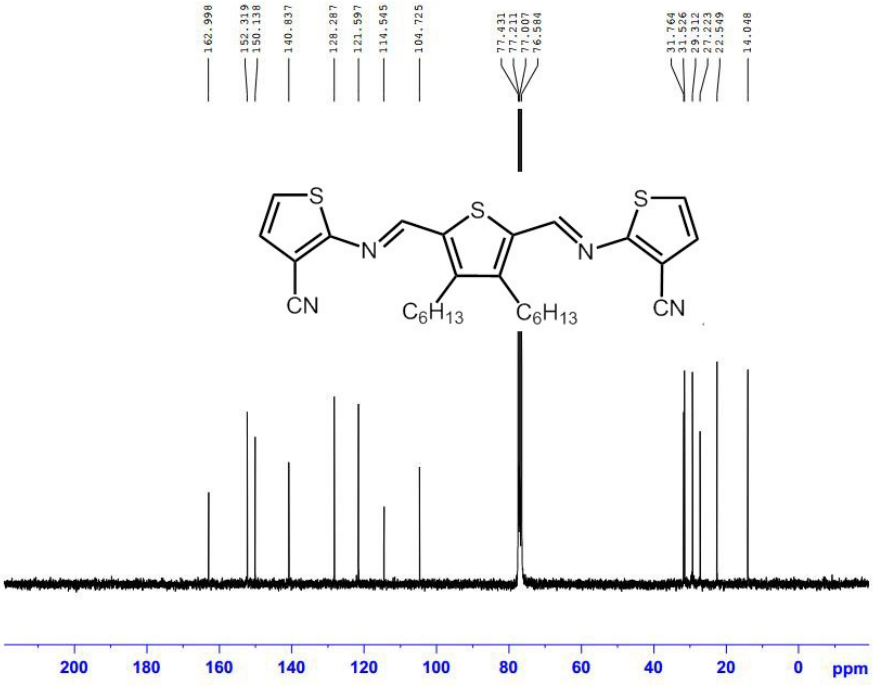

Figure S32. ^{13}C NMR spectrum of compound 11

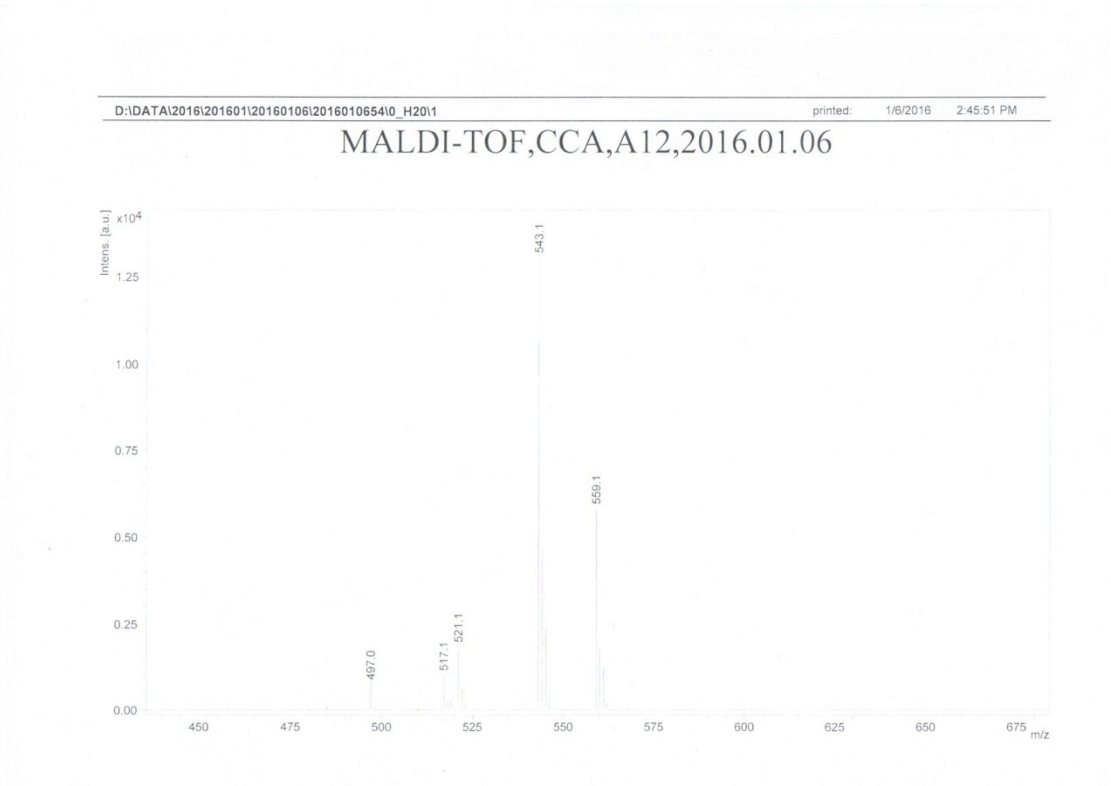

Figure S33. MS spectrum of compound 11

Synthesis of 3,4-didecylthiophene: The synthesis method was same as 3,4-dihexylthiophene. ^{1}H NMR(300MHz，CDCl3)：δ(ppm)=6.89(s,2H), 2.53-2.48(t, 4H), 1.62(s, 4H), 1.28(s, 28H), 0.89(s, 6H); ^{13}C NMR(75MHz, CDCl3)：δ(ppm)=143.2, 142.1, 119.8, 119.7, 31.9, 30.5, 30.2, 29.7, 29.6, 29.5, 29.3, 28.8, 22.6, 14.0。MS(EI-MS) calcd. for $C_{24}H_{44}S$ $[M]^+$ 364.6, found 364.

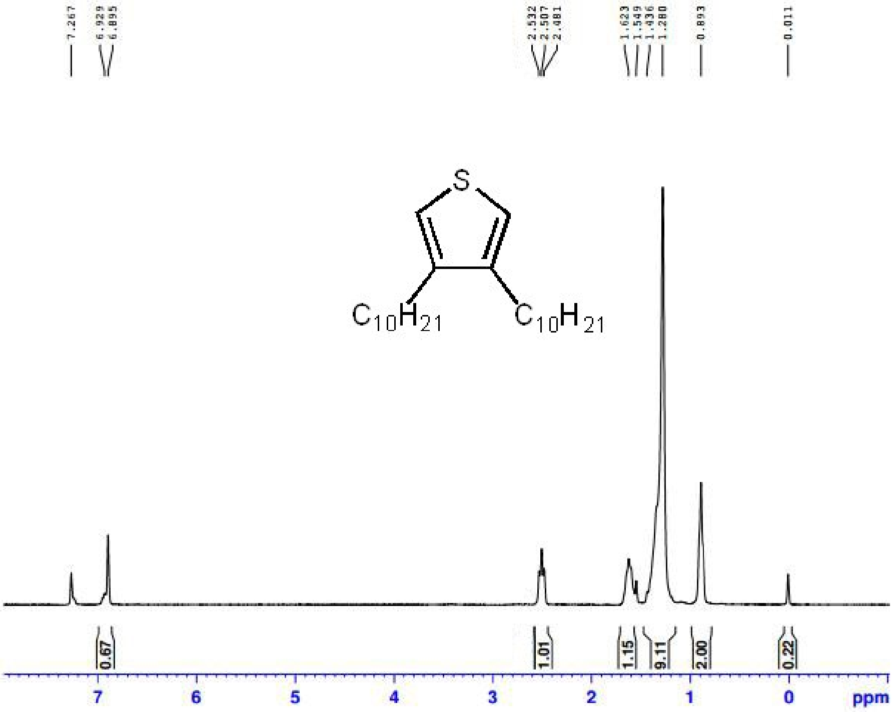

Figure S34. ^{1}H NMR spectrum of compound 3,4-didecylthiophene

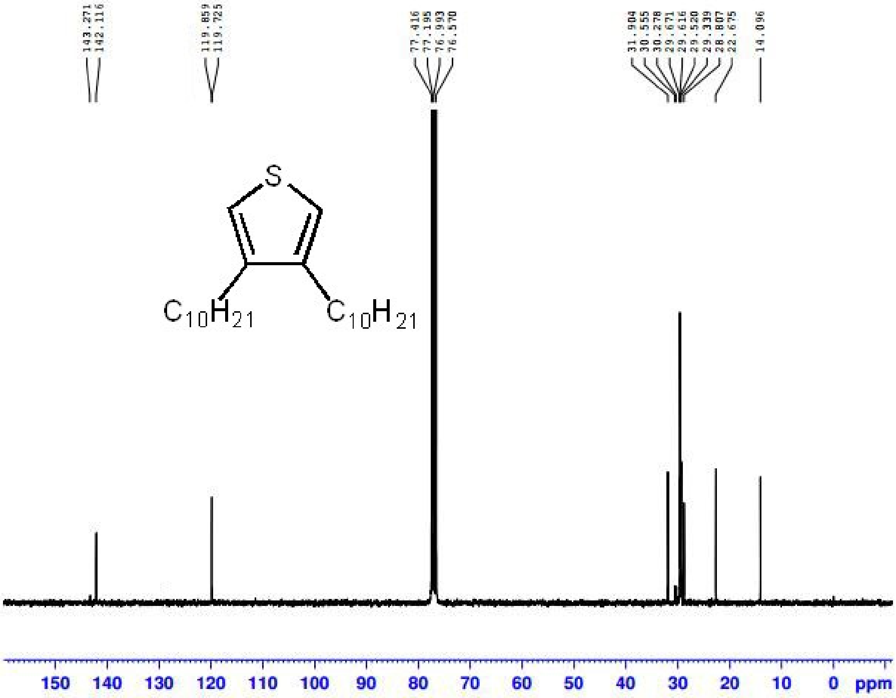

Figure S35. ^{13}C NMR spectrum of compound 3,4-didecylthiophene

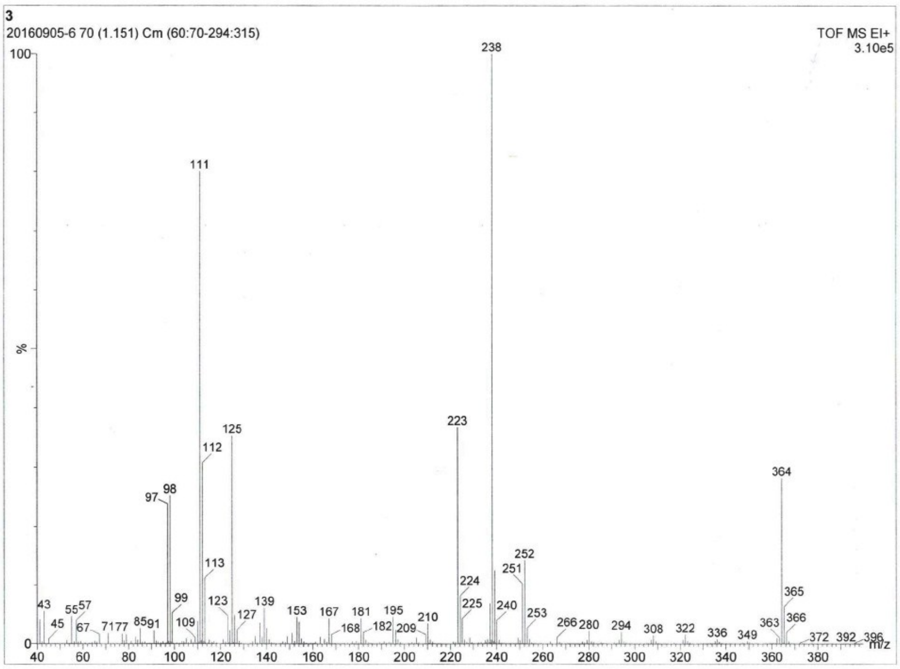

Figure S36. MS spectrum of compound 3,4-didecylthiophene

Synthesis of 3,4-didecylthiophene-2,5-dicarbaldehyde: The synthesis method was same as 3,4-dihexylthiophene -2,5-dicarbaldehyde. 1H NMR(300MHz，CDCl3)：δ(ppm)=10.12 (s, 2H), 2.93-2.88(t, 4H), 1.64-1.56(m, 4H), 1.45-1.27(m, 28H), 0.90-0.86 (t, 6H); 13C NMR(75MHz, CDCl3)：δ(ppm)=183.2, 151.6, 143.2, 32.1, 31.8, 29.6, 29.5, 29.4, 29.2, 26.6, 22.6, 14.0. MS(EI-MS) calcd. for C26H44O2S [M] + 420.7, found 420.

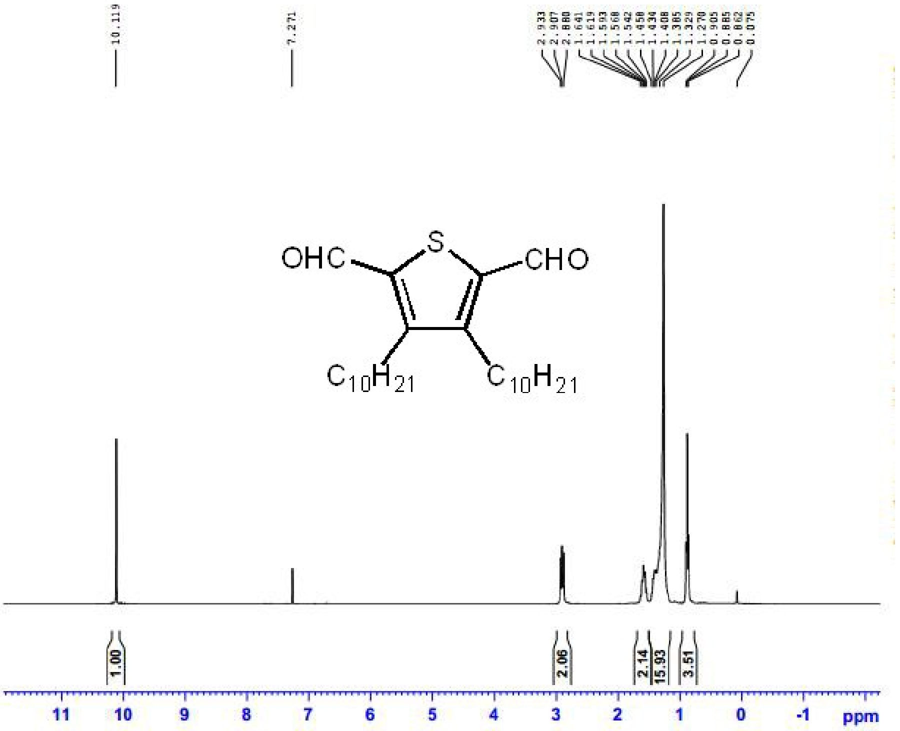

Figure S37. ^{1}H NMR spectrum of compound 3,4-didecylthiophene-2,5-dicarbaldehyde

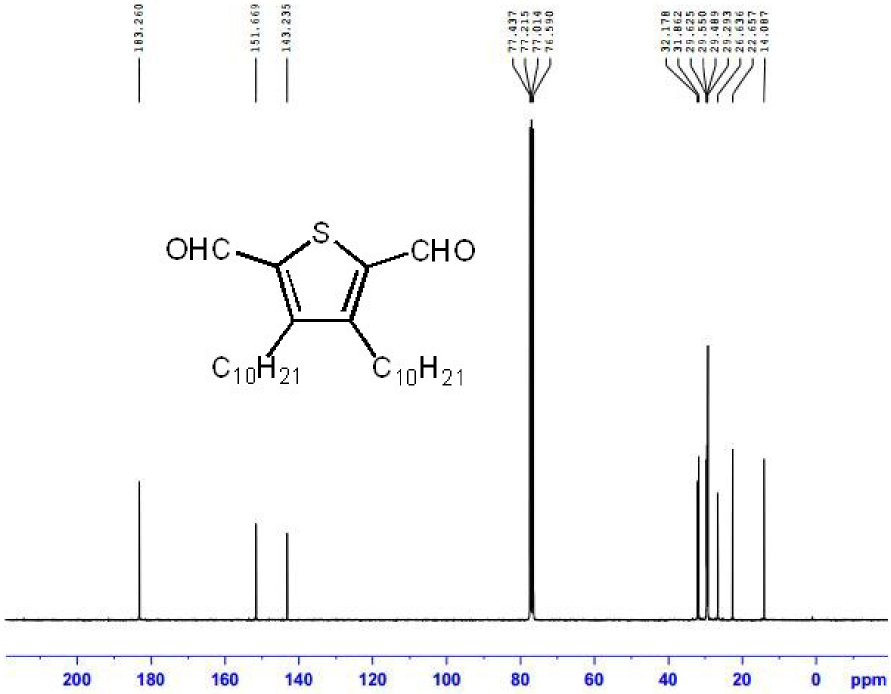

Figure S38. ^{13}C NMR spectrum of compound 3,4-didecylthiophene-2,5-dicarbaldehyde

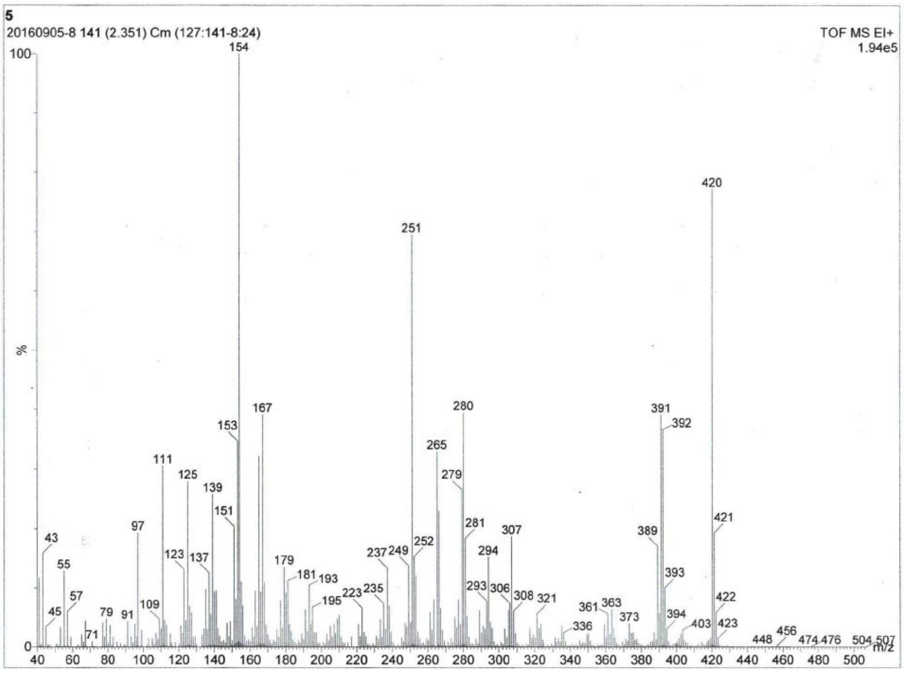

Figure S39. MS spectrum of compound 3,4-didecylthiophene-2,5-dicarbaldehyde

Synthesis of 12: The synthesis method was same as compound 11. ^1H NMR(300MHz， CDCl3)：δ(ppm)=8.69 (s, 2H), 7.14-7.11 (m, 4H), 2.85(t, 4H), 1.60(s, 4H), 1.43-1.27(m, 28H), 0.88(s, 6H); ^{13}C NMR(75MHz, CDCl3)： δ(ppm)=162.9, 152.6, 152.3, 151.7, 150.1, 140.9, 140.8, 128.2, 122.0, 121.9, 121.5, 114.5, 104.7, 31.8, 31.7, 29.6, 29.5, 29.3, 28.9, 27.2, 26.9, 25.2, 22.6, 14.1. MS(MALDI-TOF): calcd. for $C_{26}H_{48}N_4S_3$ $[M]^+$ 632.3, found 633.

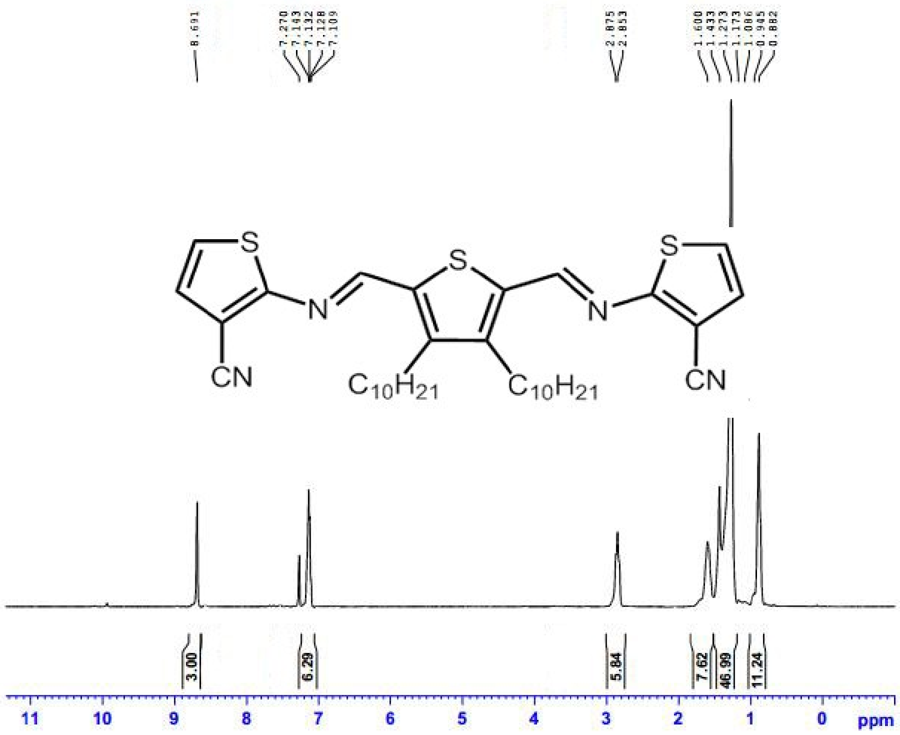

Figure S40. ^{1}H NMR spectrum of compound 12

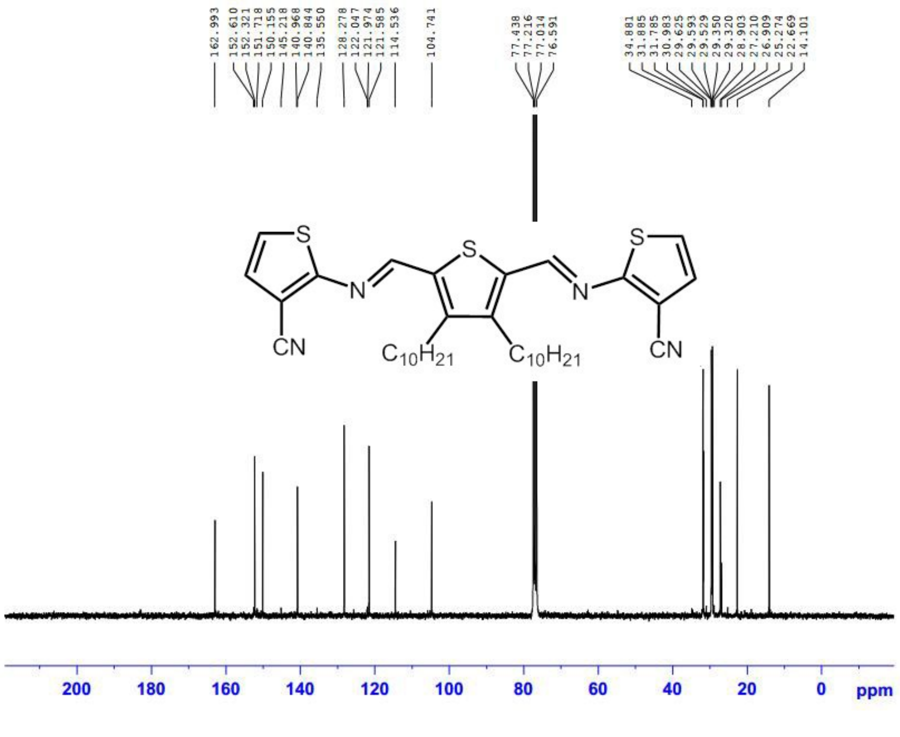

Figure S41. ^{13}C NMR spectrum of compound 12

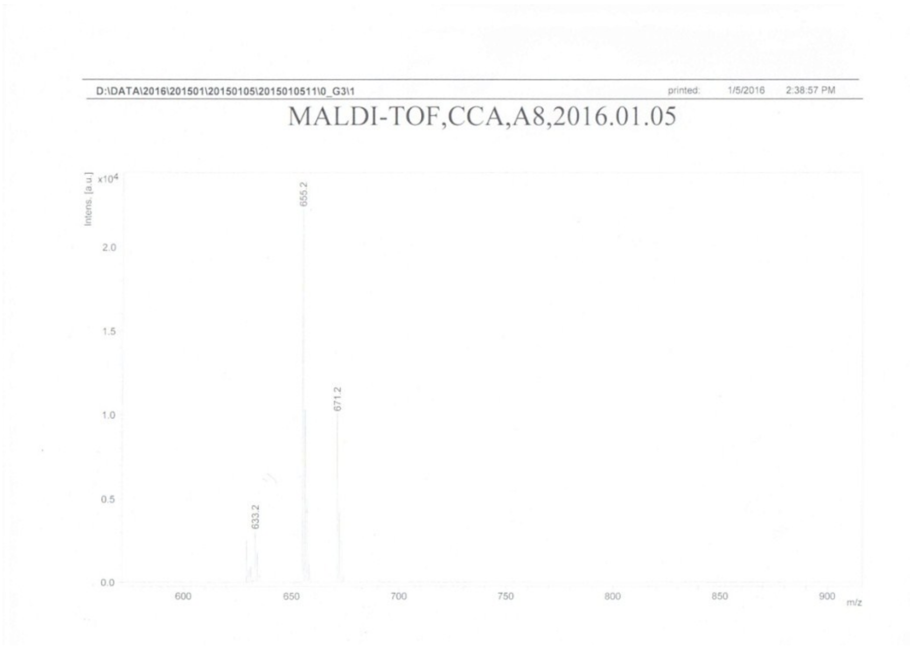

Figure S42. MS spectrum of compound 12

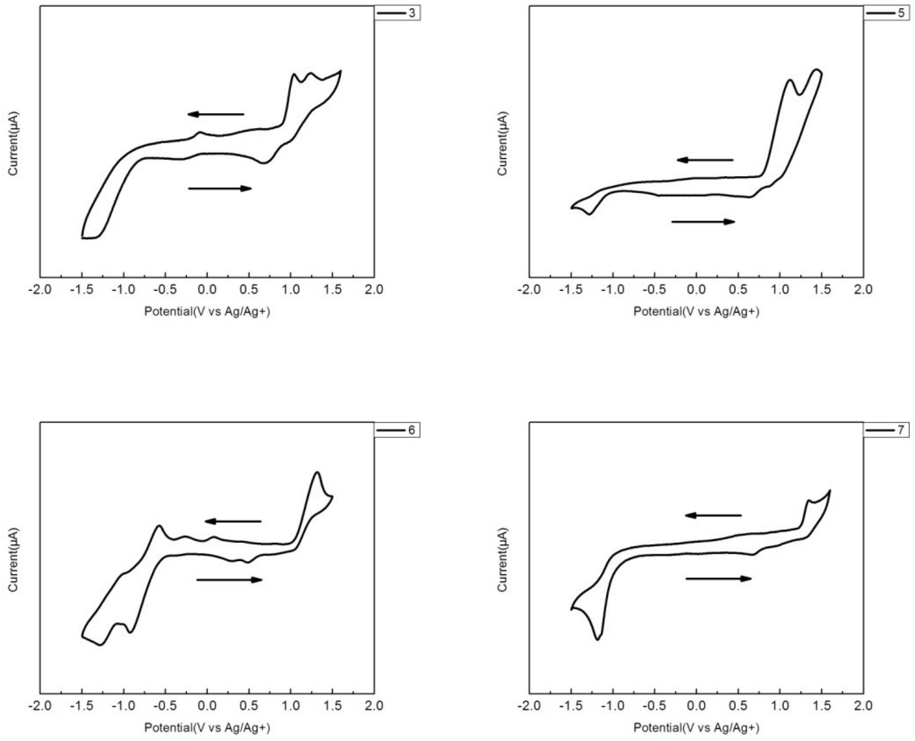

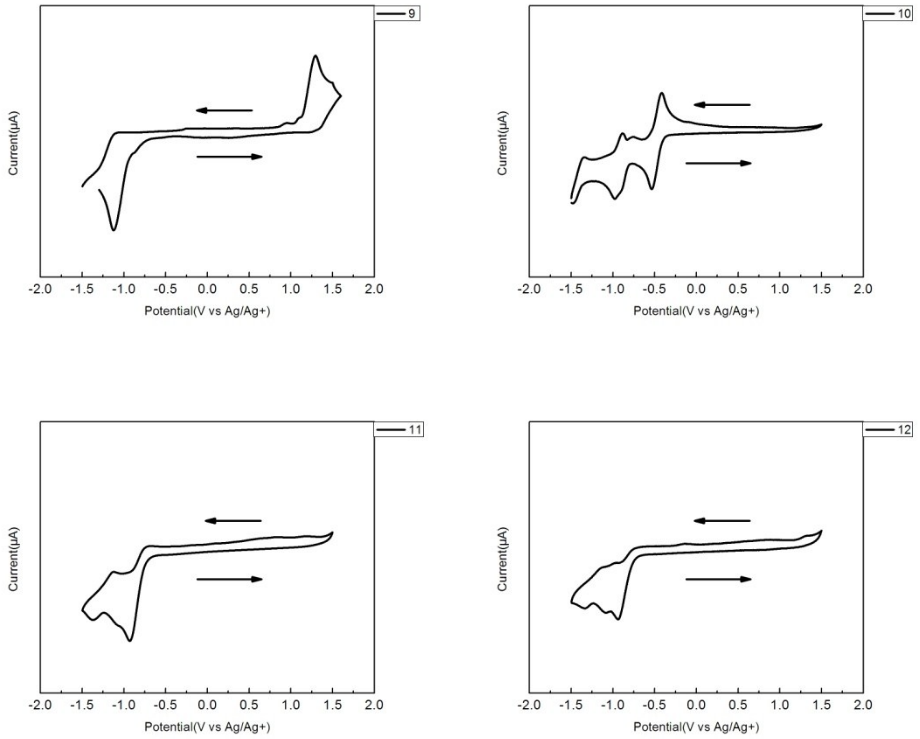

Figure S43. Cyclic voltammograms of thiopheno azomethines(3,5,6,7,9,10,11,12) at (1.0×10^{-4} M) concentration in dry dichloromethane.
